# A Review of Recent Advances in the Anticancer Mechanisms of Activity of Novel Thiazoles and 4-Thiazolidinones/Thiazolidinediones (2021–2025)

**DOI:** 10.3390/molecules31091444

**Published:** 2026-04-27

**Authors:** Rostyslav Dudchak, Magdalena Podolak, Anna Bielawska, Krzysztof Bielawski, Roman Lesyk

**Affiliations:** 1 Department of Synthesis and Technology of Drugs, Faculty of Pharmacy, Medical University of Bialystok, Jana Kilińskiego 1, 15-089 Bialystok, Poland; krzysztof.bielawski@umb.edu.pl; 2 Department of Biotechnology, Faculty of Pharmacy, Medical University of Bialystok, Jana Kilińskiego 1, 15-089 Bialystok, Poland; magdalena.podolak@sd.umb.edu.pl (M.P.); anna.bielawska@umb.edu.pl (A.B.); 3 Department of Pharmaceutical, Organic and Bioorganic Chemistry, Danylo Halytsky Lviv National Medical University, Pekarska 69, 79010 Lviv, Ukraine; 4 Molecular Design Center, Danylo Halytsky Lviv National Medical University, Pekarska 69, 79010 Lviv, Ukraine; 5 Department of Biotechnology and Cell Biology, University of Information Technology and Management in Rzeszow, St. Sucharskiego 2, 35-225 Rzeszow, Poland

**Keywords:** 4-thiazolidinone, thiazolidinedione, thiazole, anticancer activity

## Abstract

With global cancer cases projected to reach 35 million by 2050 and drug resistance to existing chemotherapeutic drugs remaining a significant threat in cancer therapy, accounting for up to 90% of chemotherapy failures, the search for novel anticancer compounds continues to be increasingly important. This systematic review (2021–2025) examined the role of thiazoles and 4-thiazolidinones/thiazolidinediones as popular scaffolds in existing anticancer drug design. While researchers continue to focus on well-established molecular targets, such as EGFR, VEGFR-2, and tubulin, there is a notable difference regarding other preferred choices for thiazoles and 4-thiazolidinones/thiazolidinediones. Among analyzed mechanisms of anticancer activity notably favored for thiazoles was the inhibition of serine/threonine protein kinases (CDK-2, BRAF^V600E^), while for 4-thiazolidinones/thiazolidinediones more studied were ROS generation and PPARγ activation. Furthermore, less-researched mechanisms of anticancer activity with no FDA-approved drugs such as PTP1B, SIRT2, PKM2, eIF4E, CA XI and XII inhibition for thiazole derivatives and pan-PIM kinase and BAG3 protein inhibition for 4-thiazolidinones/thiazolidinediones were evaluated as well. Notable was the popularity of the multi-targeting approach for modern drug design, with ~30% reporting two or more targets for their compounds. Despite these advancements, the review identified critical gaps in ADMET evaluations, safety analyzing against normal human cells and the lack of mechanistic studies connecting the targeted protein and the compounds anticancer effects.

## 1. Introduction

The thiazole (TZ) moiety is an often-utilized scaffold for the synthesis of novel organic structures with various activities due to its distinctive physicochemical properties. It is a five-membered aromatic ring with every atom being in *sp*^2^ hybridization, offering a stable, rigid and planar 6-π system with potential substitutions at C2, C4 and C5 positions. Substances with a thiazole structure have been actively utilized in medicinal practice and can be divided into the following groups: antibacterial, antiviral, anticancer, anticoagulant, antifungal, and anti-inflammatory. Antibacterial drugs include: Aztreonam, approved by the Federal Drug Association (FDA) in 1986 under the trade name Azactam^®^ [1] and in 2025 under the name Emblaveo^®^ [2], used to treat infections caused by multidrug-resistant Gram-negative bacteria. Cefdinir (Omnicef^®^) [3] was registered in 1997 for the treatment of Gram-negative bacteria, and cifiderocol (Fetroja^®^) [4] in 2019 as the first siderophore antibiotic against multidrug-resistant strains of Gram-negative bacteria, including *Pseudomonas aeruginosa*. It is utilized in treatments of complicated forms of urinary tract infections when other treatment options are unavailable. Seven substances have been registered against both Gram-positive and Gram-negative bacteria. The first of these was cefotaxime, registered under the trade name Claforan^®^ [5] in 1981 as a broad-spectrum antibiotic. Subsequently, ceftriaxone (Rocephin^®^) [6] and cefotiam (Ceradon^®^) [7] were registered in 1984 and 1988, respectively. In 1995, 1996, 2001, and 2010, the FDA approved the following substances: ceftibuten (Cedax^®^) [8], which is also used to treat enteritis and gastroenteritis; cefepime [9], which received additional registration in 2024 as a drug in combination with enmetazobactam (Exblifep^®^) [10]; cefditoren (Spectracef^®^) [11]; and, most recently, ceftaroline under the trade name Teflaro^®^ [12]. Thiazole derivatives also have proven antiviral and antifungal activity. The first antiviral thiazole derivative approved by the FDA was ritonavir (Norvir^®^) [13] in 1996 as a protease inhibitor for the treatment of HIV (Human Immunodeficiency Virus type 1)-1. The same substance was utilized in 2023 to treat the SARS-CoV-2 virus under the name Paxlovid^®^ [14]. As an antifungal, isavuconazolum, under the trade name Cresemba^®^, was approved in 2015 for the treatment of invasive aspergillosis and mucormycosis [15]. Three compounds have been registered for the treatment of gastrointestinal complaints, including the previously mentioned ceftibuten, which also has antibacterial properties. The earliest registered drug, in 1988, was nizatidine (Axid^®^) as a H_2_ receptor antagonist for the treatment of heartburn and peptic ulcers [16]. In 2002, nitazoxanide (Alinia^®^) was registered as a drug for diarrhea caused by *Gardia lamblia* and *Cryptosporidium parvum* [17]. Other effects of thiazoles include anticoagulant activity (edoxaban was registered in 2015 under the name Savaysa^®^ [18]), anti-inflammatory activity (meloxicam was registered as a COX-2 inhibitor in 2000 under the name Mobic^®^ [19]), and antihyperuricemic activity (febuxostat was registered in 2009 under the name Uloric^®^ [20]). Additionally, thiazoles are used to treat symptoms of overactive bladder with mirabegron, registered in 2012 under the trade name Myrbetriq^®^ [21], and as an acetylcholinesterase inhibitor in the treatment of Alzheimer’s disease and myasthenia gravis with acotiamide, which has not been approved by the FDA; however, it has been authorized in Japan since 2013 [22]. Among the thiazole core substances, some have also been approved for anticancer activity. The first of these was dasatinib, known as Sprycel^®^, in 2006, as a treatment for chronic myeloid leukemia, acute lymphoblastic leukemia, and Philadelphia chromosome-positive cancer [23]. Subsequently, in 2019 and 2022, alpelisib was approved under the trade names Piqray^®^ and Vijoice^®^, respectively. The former is used to treat men with locally advanced or metastatic breast cancer that is hormone receptor (HR)-positive, and human epidermal growth factor type 2 (HER-2)-negative, and for the treatment of postmenopausal women in combination with fulvestrane. Vijoice^®^, on the other hand, is used as a kinase inhibitor indicated for the treatment of adult and pediatric patients aged 2 years and older with severe PIK3CA hyperplasia spectrum symptoms who require systemic therapy [24].

Alternatively, 4-thiazolidinones (4-TZDs) and thiazolidinediones (TZDs) are saturated non-planar, flexible rings with *sp*^3^ carbons and carbonyl groups, which offer more opportunities as potential building blocks in combinatorial chemistry with multiple ring positions available for structural modifications (C2, C3, N-substitution) and especially a more synthetically available C5 position. 4-Thiazolidinone/thiazolidinedione derivatives are present in medicinal practice as well. They are primarily applied for the treatment of type 2 diabetes as a class of glitazones, which includes pioglitazone, registered under the name Actos^®^, and rosiglitazone, registered under the brand name Avandia^®^, both approved by the FDA in 1999 [25]. Lobeglitazone (Duvia^®^), used in South Korea since 2013, has not been approved by the FDA [26]. Epalerstat has been utilized for diabetic neuropathy since 1992 in Japan, China, and India; however, it was not approved by the FDA [27]. In addition to their use in the treatment of type 2 diabetes and its complications, such as diabetic neuropathy, 4-thiazolidinones have also been utilized as drugs for hypertension, as loop diuretics (etozolin is approved for medical use only in Europe under the names Elkapin^®^, Etopinil^®^, and Diulozin^®^) [28], and in the treatment of multiple sclerosis and psoriasis, for which ponesimod was approved in 2021 under the trade name Ponvory^®^ [29].

According to Bray et al., by 2050, a 77% increase in cancer cases, with 35 million new cases, with one in nine men and one in 12 women meeting a lethal outcome, is projected [30]. Furthermore, anticancer drug resistance is a significant threat to cancer treatment, with nearly 90% of chemotherapy failures directly attributed to it [31]. Consequently, the development of novel anticancer compounds remains a critical priority within medicinal chemistry. Thiazoles and 4-thiazolidinones/thiazolidinediones have been actively studied as potential anticancer compounds and may be considered as important scaffolds in the current anticancer drug design [32,33,34]. Taking into consideration the vast medical applications of these types of compounds and their structural properties, we explored the molecular targets and mechanisms of the anticancer activity of various thiazoles and 4-thiazolidinones/thiazolidinediones published in 2021–2025 (Table 1 and Table 2).

## 2. Thiazoles and 4-Thiazolidinones/Thiazolidinediones as Privileged Scaffolds in Modern Drug Design

Important requirements for potential drug candidates are optimal pharmacokinetic and pharmacodynamic profiles. To facilitate the selection of suitable compounds, the industry created a set of rules regarding ADMET (Absorption, Distribution, Metabolism, Excretion, and Toxicity) characteristics, which are based upon the currently established clinically utilized drugs and compounds in clinical trials. Those rules are applied to ensure favorable bioavailability and allow less suitable compounds to be easily filtered out during early-stage development. One of the most popular set of rules for drug-likeness was developed by Lipinski et al. [84]. According to the authors, a suitable drug candidate should have: a molecular weight less than 500 Da (Dalton), a lipophilicity (logP) less than five, a number of hydrogen bonds less than five and a number of hydrogen bond acceptors less than 10. There is an important reasoning behind every established rule. For instance, limitations on molecular weight are stated to ensure better diffusion and easier access through cell membranes. While there are exceptions with higher molecular weights, a great majority of drugs will adhere to this rule. Lipophilicity is another important factor to consider. When this parameter is too high it negatively impacts not only the aqueous solubility, but also its ability to pass through the lipid bilayer of the cell membrane. The number of groups which can donate hydrogen bonds can also negatively affect the bioavailability of compounds, since it requires more energy to break from the aqueous solvent. Alternatively, bond acceptors, such as oxygen or nitrogen atoms, increase the polarity of the compounds, negatively affecting passive diffusion through the cell membrane [85,86].

In terms of potential drug-likeness, both TZ and 4-TZD/TZD have certain advantages (Table 3). For instance, their low intrinsic molecular weight provides significant ‘headspace,’ allowing for the addition of diverse substituents without exceeding the 500 Da limit. Furthermore, the lipophilic contribution of each ring is relatively low, allowing various lipophilic substituents to be added as side chains to the ring core. The minimal number of H-bond acceptors or donors, provided by each ring, allows designing compounds with numerous active groups at side chains. These favorable physicochemical properties in combination with a high synthetic accessibility and wide range of activities confirm their status as privileged scaffolds in modern drug design.

Nonetheless, structures with those scaffolds also have certain disadvantages regarding their pharmacokinetic and pharmacodynamic profiles. For instance, thiazoles face a potential high metabolic reactivity due to present sulfur or nitrogen atoms and an aromatic ring system with many reactive positions where donor–acceptor, nucleophilic or oxidation reactions may happen and thus lead to unpredicted and undesirable side effects in the physiological systems [88]. Similarly to thiazoles, 4-TZD and TZD derivatives also face challenges related to metabolic reactivity. For instance, TZDs are extensively metabolized by CYP (Cytochrome P450) isoenzymes, particularly CYP2C8, CYP2C9, and CYP3A4, providing not only undesirable drug–drug interactions and drug–food interactions, but also causing hepatotoxicity [89]. Alternatively, 4-TZDs can sometimes be interpreted as PAINS (Pan-Assay Interference Compounds). The discussion around PAINS is controversial. Such structures may cause false positive results in various in vitro methods of analysis and showcase little selectivity towards targeted proteins [90]. Alternatively, around 5% of FDA-approved and clinically utilized drugs are identified as PAINS structures. Thus, avoiding such structures can lead to missing promising hits and leads in drug design [91,92]. Taking into consideration this information, a proper assessment of the pharmacodynamic, pharmacokinetic and safety profiles of new TZs or 4-TZDs/TZDs should be performed during the early stages of drug development.

## 3. Mechanisms of Anticancer Activity of Novel Derivatives with Thiazole Ring in 2021–2025

### 3.1. Targeting Tyrosine Kinases

#### 3.1.1. EGFR Inhibition

EGFR, also known as epidermal growth factor receptor, is one of the most overexpressed or mutated proteins in various types of cancer, such as non-small cell lung cancer; colorectal, breast, pancreatic, head, and neck cancers; and glioblastoma. In standard conditions this receptor plays an important part in promoting cell growth and preventing apoptosis. By targeting EGFR, several outcomes in terms of anticancer activity can be obtained from numerous downstream signaling pathways. One of the main signaling cascades which will be affected is the RAS-RAF-MEK (Mitogen-Activated Protein Kinase)-ERK (MAPK) cascade. This pathway promotes proliferation, differentiation, and cell growth. By inhibiting EGFRs, the MAPK (Mitogen-Activated Protein Kinase) cascade will be hindered and, as a result, the active multiplication of cancerous cells will be impeded. Another outcome of EGFR suppression is the stimulation of the process of programmed cell death. Cancer cells utilize the PI3K-AKT (Protein Kinase B)-mTOR pathway to prevent apoptosis through the inhibition of pro-death proteins: BAD (Bcl-2-associated death promoter) by phosphorylation and BIM (Bcl-2 Interacting Mediator of cell death) by preventing its synthesis. However, the inhibition of EGFR will lead to the blockade of this signaling cascade, restoring BAD activity and BIM levels and, as a result, promoting apoptosis [93,94]. The Jak (Janus kinase)-STAT (Signal Transducers and Activators of Transcription) pathway in cancer cells is responsible for gene transcription, which ultimately leads to proliferation, cell growth, immune response, and metastasis. Cytokines, hormones, and growth factors are responsible for activating the pathway. The binding of a cytokine, hormone, or growth factor to a receptor outside the cell leads to the phosphorylation of Jak, which in turn leads to the phosphorylation of a single STAT molecule. Through phosphorylation, two STAT molecules bind together and, in this form, can enter the cell nucleus, where they bind to the promoter region and regulate transcription [95,96].

**Figure 1:**
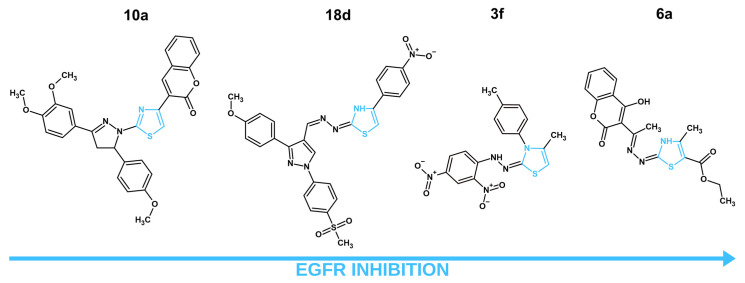
The structures of thiazole derivatives targeting EGFR inhibition published in 2021–2025. Compound names are preserved from their source publications for consistency.

In recent years, four articles showcased thiazole derivatives targeting EGFRs as potential anticancer agents (Table 4; Figure 1). Structural preferences for better EGFR inhibition were noted. For instance, a bulky multi-heterocyclic system containing pyrazoline and pyrazole was connected to the C2 position of more potent EGFR inhibitors **10a** and **18d**, respectively. In the case of the strongest EGFR inhibitor **10a**, two methoxyphenyl groups were attached to pyrazoline. Methoxyphenyl group connection to pyrazole was also present in **18d**. However, the second substitute was different; the phenyl-methylsulfonyl group was likely present to allow for unique interactions with otherwise unavailable mutated forms of EGFR. Dinitrophenyl and hydroxycoumarin moieties were present at the C2 positions of less active EGFR inhibitors **3f** and **6a,** respectively. Interestingly enough, **18d**, **3f** and **6a** had an azine or hydrazone bridge before their respected substituents at the C2 position. However, it does not seem to impact the EGFR inhibition in significant way as the strongest EGFR inhibitor **10a** had a direct connection at the C2 position. The N3 position of the thiazole core remained mainly untouched, except for **3f**, which had a *p*-tolyl substituent at this position. Alternatively, the C4 position of the thiazole core seems to have a significant impact on EGFR inhibitory activity. While the less active **3f** and **6a** had a simple methyl group, more effective compounds showcased more complex aromatic derivatives, such as coumarin (**10a**) and *p*-nitrophenyl (**18d**). Interestingly enough, the most potent and the least potent EGFR inhibitors shared the coumarin fragment. However, the positioning seems to play a significant role, as the C4 position and direct connection showcased more promise in comparison to the C2 position and bridged connection. Lastly, the C5 position remained mostly untouched, except for **6a**, which had ethyl carboxylate.

In a manuscript published by Al-Wahaibi et al., the authors presented nine novel 2,3,4-trisubstituted thiazole derivatives (**3a–3i**), which were tested against breast (MCF-7), lung (A-549), pancreatic (Panc-1) and colon (HT-29) cancer cell lines for their antiproliferative capabilities [40]. The most potent compound was **3f** which displayed similar IC_50_ concentrations on all cell lines with an average equal to 0.037 µM. Next, compounds **3a**, **3c**, **3d** and **3f** were analyzed for their EGFR inhibitory capabilities. Compound **3f** continued the trend, showcasing the most promising results with the IC_50_ of EGFR inhibition equal to 0.089 µM, which was comparable to the reference drug—erlotinib, with IC_50_ equal to 0.080 µM. The authors also tested their compound for BRAF^V600E^ kinase inhibition to display the dual-targeting mechanism of the novel compounds. Compound **3f** exhibited the lowest IC_50_ = 0.093 µM. The lack of an ADMET properties assessment of novel structures should be noted. Such information is crucial before proceeding further in the drug development process. Other problems, such as insufficient mechanistic studies and the absence of in vitro testing against normal cells, were observed. Although the obtained data shows promise, a more in-depth analysis of the compound’s mechanism of activity and its safety, pharmacokinetic and pharmacodynamic properties should be performed in future research. In another article by Fakhry et al., a series of thiazolyl-pyrazoline derivatives (**4a–d, 5a–d, 6a, b, 7a–d, 8a, b,** and **10a, b**) were synthesized with anticancer potential through a dual mechanism of activity with EGFR/HER-2 inhibition in mind [41]. All of the new structures were tested for their cytotoxic activity after 48 h of treatment against MCF-7 breast cancer cells. The obtained results showcased that compounds **6a, 6b** and **10a, 10b** exhibited the lowest IC_50_, equal to 4.08, 5.64, 3.47 and 3.54 µM, respectively. The most potent compounds were further tested against normal breast cells (MCF-10A) to analyze the compounds’ selectivity towards cancer cells. The structures **6a** and **10a** did not exhibit toxicity towards this cell line, even at 50µM, while the **6b** and **10b** IC_50_ values were equal to 32.81 and 27.14 µM, respectively. In terms of EGFR inhibition, compound **10a** exhibited more prominent results with IC_50_ inhibition equal to 0.005 µM, which was in close similarity to the reference drug—lapatinib, with 0.007 µM. Compound **10a** also displayed the lowest IC_50_ required for HER-2 inhibition, equal to 0.022 µM. A cell cycle assessment in MCF-7 cells for compound **10a** did not showcase a significant difference in comparison to the control. However, an apoptosis analysis provided interesting results, where compound **6a** exhibited 51.03% of apoptotic cells, while **10a** had only 42.66% apoptotic cells. Such data confirms the potency of the **10a** compound with a coumarin substitute at C4 of the thiazole pharmacophore, but it also may suggest that compound **6a** will have another molecular target with proapoptotic potential. The ADMET properties of the synthesized compounds were analyzed via an in vitro approach. Compound **10a** showcased one violation of Lipinski rules regarding its poor aquesous solubility, which also leads to predicted poor GIT absorption. Comparatively, **6a** did not exhibit any violations of Lipinski rules with superior solubility and better GIT absorption. Both compounds showcased no penetration through blood–brain barrier (BBB). Compound 10a displayed no PAINS alert. Despite potent anticancer activity and a promising safety profile, compound **10a**’s predicted GIT absorption and poor solubility are concerning. Perhaps further structural modifications to improve those characteristics are needed. The multiple target mechanism of anticancer activity was also explored by Fadaly et al. [42]. In their manuscript, the authors presented two new series of compounds with a pyrazole core with a methylsulfonyl group and either phenylthiazole (**18a–j**) or thiazolidinone (**16a–b**) connected via a hydrazine linker. Novel compounds were designed to target EGFR, COX-2 and HER-2. An MTT assay was performed to identify the most potent compounds and to analyze their safety towards normal cells. The compounds were tested against MCF-7, A-549 and F180 (human normal fibroblast cells). Among the tested thiazole derivatives, compounds **18c, d** and **f** showcased the most promising results among thiazole derivatives on the MCF-7 cell line, with IC_50_ values ranging from 3.18 to 2.15 µM. The selectivity index for those were quite satisfactory as well, varying between 8.92 and 18.72. Most interesting were the results of EGFR inhibition. The authors tested compounds **16a, 18c**, **18d** and **18f** against three different EGFR kinases: EGFR^wt^, EGFR^L858R^ and EGFR^L858R/T790M^. EGFR^wt^ stands for wild-type epidermal growth factor receptor, a normal functional version. On the other hand, EGFR^L858R^ and EGFR^L858R/T790M^ are both mutations often present in cancer. EGFR^L858R^ mutation causes the EGFR to be constantly active, driving continuous cell proliferation. The EGFR^L858R/T790M^ not only has the previously described mutation but also a T790M mutation known to provide increased resistance to numerous anticancer drugs [97]. By testing synthesized compounds against all three types of EGFR, the authors wanted to showcase the safety and efficacy of their structures. Compound **18d** displayed extraordinary results with a 6.12 selectivity index, calculated by dividing the IC_50_ for EGFR^wt^ by the IC_50_ for EGFR^L858R^ and 2.37 for EGFR^wt^/EGFR^L858R/T790M^. For comparison, the reference compound, an established anticancer drug, Dasatinib, exhibited only 2.05 and 1.35 selectivity indexes for EGFR^wt^/EGFR^L858R^ and for EGFR^wt^/EGFR^L858R/T790M^, respectively. To provide further evidence of the anticancer effects of structure **18d**, cell cycle analysis and apoptosis assessments were performed on MCF-7 cells. While the cell cycle results did not showcase a significant difference between the cells treated with **18d** and controlled cells, an apoptosis assessment provided promising results, with **18d** increasing the population of apoptotic cells by 39.32% in comparison to the control. It is worth mentioning that a thiazolidinone derivative **16a** showcased superior anticancer activity (IC_50_ = 0.73 µM) and selectivity (SI = 33.15) on MCF-7 cells. Furthermore, this compound displayed more exceptional effects in both COX-2 and HER-2 inhibitory assays. Compound **16a** exhibited extraordinary selectivity towards COX-2, with the IC_50_ required to inhibit this protein equal to 0.349 µM, while the IC_50_ for COX-1 was 46.98 µM. Thus, this compound possessed a selectivity index for COX-2/COX-1 equal to 134.6, which was notably higher than both thiazole derivative **18d** (SI = 2.629) and the reference compound, the selective COX-2 inhibitor celecoxib (SI = 24.09). A HER-2 inhibitory assay showcased similar results to that of COX-2 inhibition, with compound **16a** showcasing an IC_50_ equal to 0.032 µM in comparison to compound **18d** (IC_50_ = 0.195 µM) and comparable to reference compound dasatinib (IC_50_ = 0.041 µM). The ADMET properties of new structures were analyzed utilizing the in vitro method. Compound **18d** displayed two violations of Lipinski rules, poor aquesous solubility, GIT absorption and overall bioavalibility. Compound showcased more lipophilicty than the reference compound (dasatinib). Comparatively, **16a** exhibited no violations of Lipinski rules and no ability to penetrate the BBB. This compound showcased promising GIT absorption and lipophilicity and water solubility identical to the reference compound (dasatinib). Taking into consideration the analyzed data, it is to our belief that compound **18d** requries further structural improvements to adress its less favorable ADMET properties. Alternatively, **16a** exhibited a better ADMET profile and should be further researched as a promising HER-2 and COX-2 inhibitor. Similarly to the previously described article, a series of novel thiazole–coumarin derivatives were synthesized by Batran et al. [43]. Novel compounds were tested against MCF-7, HCT-116 (colon cancer), and HepG2 (liver cancer) cancer cell lines and a normal fibroblast BJ-1 cell line. In consideration with the MTT assay results, compounds **2a–b** and **6a–b** displayed the most potent activity on the MCF-7 cell line and were chosen for further analysis. Those new thiazole derivatives were tested against EGFR, mTOR and PI3K proteins via respective inhibitory assays. The structure with unsubstituted ethyl carboxylate—**6a**—displayed the most promising IC_50_, equal to the 0.184 µM required for EGFR inhibition. This structure remained the most potent against other tested proteins, with IC_50_ for mTOR = 0.719 µM and PI3K = 0.131 µM. Such data suggests a direct link between the EGFR inhibition caused by compound **6a** and its effects on the PI3K/mTOR signaling pathway. This connection was described previously as one of the main cascades of signals affected by EGFR inhibition. Next, **6a**’s effects on cell cycle and apoptosis induction were analyzed. Compound **6a** displayed a notable increase in cell population in the S phase in comparison to the untreated control. The new structure also induced apoptosis in MCF-7 cells after 48 h of treatment, up to 31.94% in comparison to control cells with only 0.67%. An in vitro assessment of the ADMET properties of **6a** displayed promising results. Compound **6a** did not violate Lipinski rules, had zero PAINS alerts, no ability to penetrate the BBB and a favorable bioavailibility score. While **6a** exhibited advantegous ADMET properties and in vitro results as a multi-target anticancer compound, the lack of safety evaluation against normal cells is a significant issue that should be adressed in future research.

#### 3.1.2. VEGFR-2 Inhibition

Another actively researched tyrosine kinase as the potential molecular target for thiazole derivatives in the last five years was VEGFR-2. It is a well-known tyrosine kinase receptor that could be the most frequently observed on endothelial cells. The main function of VEGFR-2 is to receive signals from the VEGF-A (vascular endothelial growth factor A) protein to start the process of angiogenesis, also known as blood vessel formation. Similarly to the effects of EGFR inhibition, VEGFR-2 also affects the MAPK pathway. However, VEGFR-2 mainly affects endothelial cells, causing them to no longer multiply, thus preventing the formation of new blood vessels, necessary for the survival and further growth of the tumor. Comparatively as EGFR inhibitors, compounds that impede VEGFR-2 also hinder the PI3K/AKT signaling cascade that promotes cell survival and prevents the process of programmed cell death. Blocking this pathway will naturally promote apoptosis in endothelial cells, reducing the tumor growth, survival, and its capability to migrate [98,99]. Differently from EGFR inhibition, VEGFR obstruction can cause a reduction in vascular hyperpermeability caused by an increase in eNOS (endothelial nitric oxide synthase) and ERK1/2 in endothelial cells. This decreases the so-called “leakiness” of blood vessels exploited by tumors, further reducing their capability to gather nutrients for growth [100]. However, this can also cause the lesser effectiveness of combinational therapy, in which other anticancer drug delivery could be less active due to the decreased permeability of endothelial cells. A study previously reported that in this case, the timing and dosage of VEGFR inhibitors is crucial, because a temporal increase in delivery and efficacy of drugs during the “normalization window”, a period when this effect could be utilized to obtain increased effects for various compounds, is possible [101]. Without a doubt, VEGFR-2 tyrosine kinase inhibition provides significant potential for anticancer treatment.

**Figure 2:**
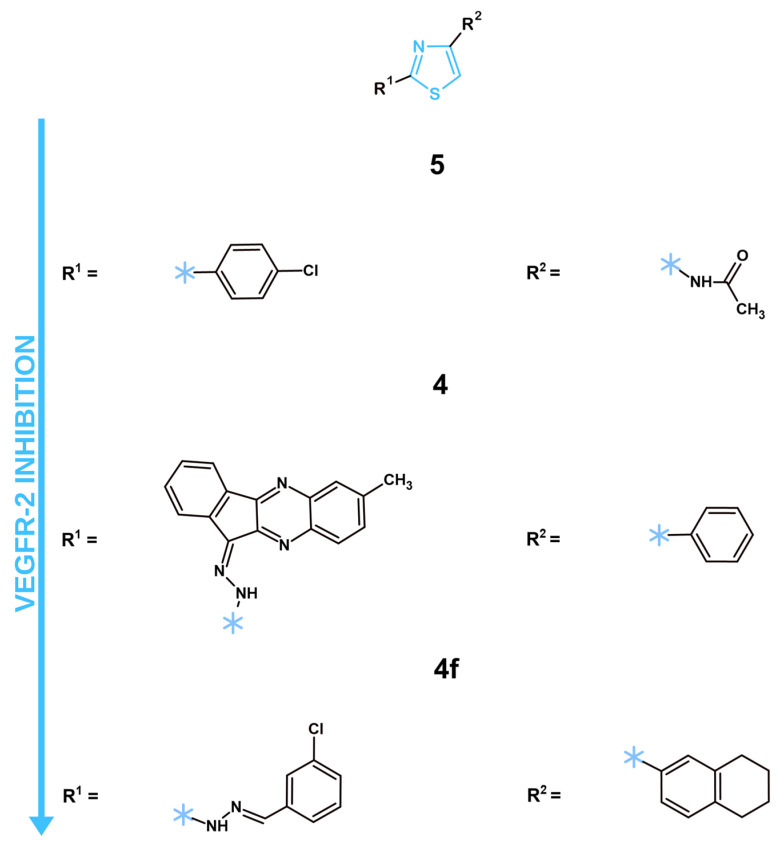
The structures of thiazole derivatives targeting VEGFR-2 inhibition published in 2021–2025. The compound names are preserved from their source publications for consistency.

In recent years, three articles showcased synthesized and tested novel VEGFR-2 inhibitors with a thiazole ring (Table 5; Figure 2). A structural analysis of the most potent VEGFR-2 inhibitors with the thiazole ring revealed that a simple 4-chlorophenyl directly connected to the C2 position of the thiazole core, present in compound **5**, exhibited better effects than the hydrazone-connected substituents observed in **4** and **4f.** Interestingly enough, a more complex and bulky indeno-quinoxaline exhibited better effects in comparison to 3-chlorophenyl. Perhaps a certain rigidness is more beneficial at this position. Another important position is C4 of the thiazole core. The most active VEGFR-2 inhibitor, compound **5**, showcased an acetamide group in comparison to the less active **4** and **4f**, which displayed cyclic systems, such as phenyl and 5,6,7,8-tetrahydronaphtalene, respectively. The higher activity of compound **5** suggests that small, polar groups capable of forming hydrogen bonds are more favorable at the C4 position rather than large, non-polar carbocycles. While increased hydrogen-bonding capability enhances potency, it should be noted that this may also cause potential side effects through undesirable interactions with other proteins. Other positions, such as N3 and C5 of the thiazole core, remained unsubstituted in all three compounds.

In the article by Salem et al. published in 2022, the authors synthesized eight new structures with a thiazole ring with anticancer potential [44]. All of the novel compounds were tested via MTT analysis against breast cancer cells (MCF-7, MDA-MB-231) and normal breast cells (MCF-10A) in comparison to staurosporine after 24 h of treatment. Among the analyzed structures, the compound with an acetyl group connected via amine to the C2 position of the thiazole core—**4**—displayed the most promising results with IC_50_ values equal to 5.73 and 12.15 µM for MCF-7 and MDA-MB-231, respectively. Moreover, the IC_50_ of this structure against normal breast cells was 36.83 ± 0.50 µM, which is around 3–6 times more than on tested cancer cells. Next the authors tested the compound **4** capability to inhibit the main molecular target—VEGFR-2. Structure **4** displayed a strong inhibitory effect on VEGFR-2 with an IC_50_ equal to 0.093 µM. Such results are only slightly worse than the reference drug—sorafenib, with an IC_50_ equal to 0.059 µM. Other methods of research, such as cell cycle and apoptosis assessment, were performed to further analyze the anticancer properties of the compound **4** on MCF-7 cells. This new structure showcased its capability to notably increase the pre-G1 phase and decrease the G2/M phase of the cell cycle of MCF-7 cells. Furthermore, compound **4** displayed 19.89% of total apoptotic cells, a notable difference in comparison to the control with 0.86% of apoptotic cells. Despite promising in vitro results against cancer cells, optimal safety and selectivity, the lack of an ADMET properties assessment should be noted. An analysis of the pharmacokinetic and pharmacodynamic properties of compound **4** has to be performed in future research. Another article by Abusaif et al. [45] presented seven new indeno[1,2-*b*]quinoxaline derivatives with anticancer potential, among which six included a thiazole ring. All the newly synthesized compounds were tested with the MTT assay against liver cancer HepG2, HuH-7, and normal immortalized liver THLE-2 cells to determine their cytotoxicity and selectivity towards cancer cells after 48 h of treatment. The most promising structure was compound **5** with a *p*-tolyl substitute connected to the C3 atom of the thiazole ring, which displayed IC_50_ values equal to 0.75 and 3.43 µM on HepG2 and HuH-7 respectively. The selectivity indexes of this structure ranged from 7.43 to 34.22, showcasing a promising safety profile. The results of VEGFR-2 inhibition were satisfactory as well, with an IC_50_ for VEGFR-2 inhibition equal to 0.061 μM, a result comparable to the reference compound—Sorafenib with 0.059 μM. The authors further confirmed compound **5**’s capability to decrease VEGFR-2 concentration in HepG2 cells after 48 h of treatment by 38.32% at its IC_50_ concentration utilizing an ELISA (Enzyme-Linked Immunosorbent Assay). This compound reduced the phosphorylated VEGFR-2 concentration by 77.64% in comparison to the untreated control. Moreover, compound **5** also displayed its potent proapoptotic effects by increasing the amount of total apoptotic cells by 44.55% in comparison to the control. Further confirmation of its proapoptotic capabilities was presented in the form of increased protein expressions of the proapoptotic proteins Bax and caspase-3, while at the same time having a reduced gene expression of the anti-apoptotic Bcl-2 protein. The authors also explored the concentration of the AKT protein, which, as previously mentioned, plays an important role in cancer cell survival mechanisms. Compound **5** decreased the total AKT by 55.29% and the phosphorylated AKT by 78.01%. The decrease in phosphorylated AKT was remarkably similar to phosphorylated VEGFR-2, which might be a consequence of VEGFR-2 inhibition. Despite promising anticancer effects, favorable selectivity and interesting results regarding the mechanism of activity, the lack of a pharmacokinetic and pharmacodynamic properties assessment was observed. ADMET properties analysis remains a crucial step in modern drug design and without it, further steps cannot be performed. Thus, such assessment should be performed in future research. In another article, authors presented 13 novel tetralin derivatives with a thiazole ring [46]. All new structures were evaluated against breast cancer (MDA-MB-231, MCF-7) and prostate cancer (PC-3) cell lines to identify the most active compound. Among those tested, compound **4f** showcased the highest IC_50_ equal to 40.2 µM against the MDA-MB-231 cell line. This correlated with the results of VEGFR-2 inhibition, where this particular compound displayed superior results with an IC_50_ equal to 0.114 µM, which was only slightly higher than the reference compound—sorafenib with 0.090 µM. Next, the authors measured the effects of **4f** on the induction of apoptosis. The obtained data showcased a notable increase in the total population of apoptotic cells up to 20.83% in MDA-MB-231 after 24 h of treatment at the IC_50_ concentration. Further confirmation of the proapoptotic effects of the chosen compound was done by measuring the gene expressions of key proapoptotic proteins (cytochrome C and Bax) and anti-apoptotic Bcl-2. Predictably, **4f** significantly increased cytochrome C and Bax expressions, while distinctively decreasing Bcl-2 expression. An ADMET properties analysis was performed via in vitro approach. New compounds exhibited only one Lipinski rule violation in terms of more than desirable lipophilicity. The lack of CNS (Central Nervous System) penetration and favorable GIT absorption were observed for **4f**. However, this compound exhibited the possibility to become a substrate for P-glycoprotein, which can negatively impact its activity and overall bioavailability. In conclusion, **4f** showcased a notable inhibition of the VEGFR-2 molecular target. However, its ADMET properties, high IC_50_ concentrations and the lack of in vitro/in vivo safety assessment provide significant challenges for further drug development.

### 3.2. Targeting Serine/Threonine Protein Kinases

#### 3.2.1. CDK-2 Inhibition

**Figure 3:**
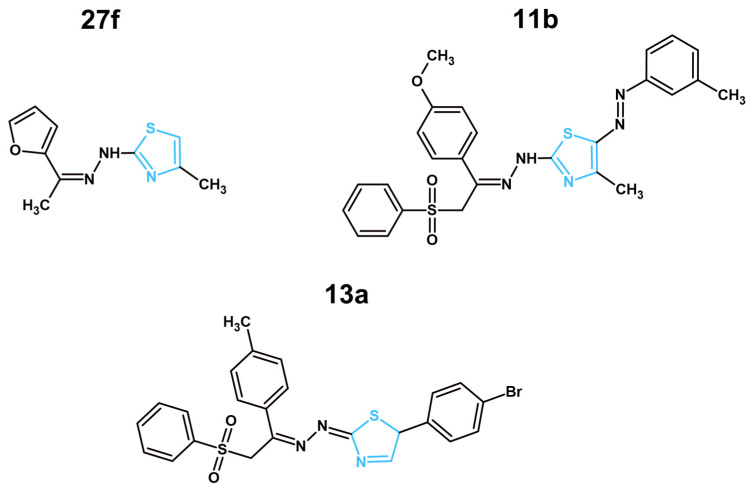
The structures of thiazole derivatives targeting CDK-2 (**27f, 11b**) and BRAF^V600E^ (**13a**) inhibition published in 2021–2025. The compound names are preserved from their source publications for consistency.

Another targeted kinase in recent years was CDK-2, with two articles showcasing novel structures targeting this protein (Figure 3) [47,48]. CDK-2 plays a critical role in cell proliferation and cell cycle progression, especially in the case of blocked CDK-4/6. The overexpression of Cyclin E, which then binds to CDK-2, induces the cell cycle transformation to S-phase, causing active DNA replication. This “escape route” allows cancer cells to overcome the effect of the anticancer drugs targeting CDK-4/6, which makes CDK-2 an important molecular target in modern anticancer research, as drugs affecting this target can be used as a part of combined therapy together with CDK-4/6 inhibitors to amplify the effects of the latter and prevent the acquired resistance towards those drugs [102,103]. In an article presented by El-Naggar et al., a series of novel thiazole–hydrazynil derivatives (**27a–r**) were designed to mimic hydrogen bonding interactions of the established CDK-2 inhibitor, roscovitine, with hydrazinyl-thiazole expected to interact with the kinase hinge region. Moreover, novel structures were synthesized utilizing a unique one-pot method of synthesis in ultrasound and microwave conditions, showcasing the eco-friendly approach of the authors [47]. Novel compounds were tested against the HepG2, MCF-7, and HCT-116 cancer cell lines, and normal fibroblast WI-38 cells. All the synthesized compounds were also analyzed for their inhibitory effects on CDK-2, CDK-1, and EGFR molecular targets. Based on the results of those two research methods, structure **27f** was observed as the most promising compound in this manuscript due to its potent activity against the HepG2 cell line (IC_50_ = 8.49 µM), safety with IC_50_ = 73.45 µM against normal WI-38 cells and extraordinary selectivity towards CDK-2 (IC_50_ = 0.35 µM) in comparison to CDK-1 (IC_50_ = 31.63 µM) or EGFR (IC_50_ = 99.82 µM). This compound was further researched for its effects on cell cycle and apoptosis induction in HepG2 cells after 48 h of treatment. The obtained data showcased that compound **27f** arrested the cell cycle in the G2/M phase, similarly to the reference compound, roscovitine, an established CDK-2, 7 and 9 inhibitor. This further confirmed the selective activity of **27f**. Moreover, this compound increased the amount of total apoptotic cells in HepG2 cells after 48 h of treatment up to 24.79%, a comparable result to roscovitine with 29.98%. While the authors reported promising anticancer activity, favorable selectivity towards cancer cells and targeted protein, a lack of ADMET assessment was observed. Without a detailed analysis of the pharmacokinetic and pharmacodynamic properties of the new compounds, next steps in drug design cannot be performed. Thus, the assessment of ADMET properties should be performed in future works. Another study by Farghaly et al. showed novel thiazole derivatives with a phenylsulfonyl moiety as potential CDK-2 inhibitors [48]. The new compounds were based on a previously described compound (**IV**) with a thiazole ring. The reasoning behind the addition of a phenylsulfonyl fragment was to extend the existing structure, provide hydrogen bonding with Lys89 residue in the CDK-2 active site and create hydrophobic interaction with the Leu298 residue in the hinge region of the molecular target. Such modifications were designed and tested with the use of molecular docking. Next, the newly synthesized compounds were assessed against HepG2, MCF-7, and PC-3 cancer cells. Among them, **11b** displayed the most potent activity against HepG2 (IC_50_ = 0.111 µM) and MCF-7 (IC_50_ = 0.245 µM) cells. This result correlated with their inhibitory effects on CDK-2 protein. Compound **11b** displayed the lowest IC_50_ (0.416 µM) among the tested compounds, comparable to roscovitine (0.432 µM). However, further research of the compound’s ADMET properties, its effects on the cell cycle, apoptosis induction, or cytotoxicity effects on normal cells was not performed, raising certain concerns regarding the safety and selectivity of this compound. Moreover, the IC_50_ of **11b** on HepG2 and MCF-7 cells was lower than the required concentration for CDK-2 inhibition. This may suggest that **11b** will affect other molecular targets at lower concentrations.

#### 3.2.2. BRAF^V600E^ Inhibition

Another article with thiazole derivatives containing a phenylsulfonyl moiety was published by Khormi et al. (Figure 3) [49]. In this manuscript, the authors displayed their newly synthesized structures targeting a BRAF^V600E^ mutated kinase. This protein is an established molecular target in melanoma treatment, as it is the most common mutation for this type of cancer. BRAF plays a critical role in the MAPK signaling pathway by phosphorylating MEK. The V600E mutation causes the BRAF kinase to be continuously active, signaling the cell to divide uncontrollably, leading to cancer. Since there is an FDA-approved thiazole derivative, dabrafenib, which selectively targets BRAF^V600E^, it is logically consistent to research novel compounds with a similar mechanism of action [104]. In the article by Khormi et al., the authors tested their newly synthesized compounds for their effects against BRAF^V600E^ and BRAF^WT^ kinases utilizing an in vitro inhibitory assay to analyze the selectivity of those compounds for their molecular target. Compound **13a** exhibited the lowest IC_50_ towards BRAF^V600E^, equal to 0.023 µM, which was nearly two times lower than dabrafenib (IC_50_ = 0.047 µM). Other compounds, such as **7b** and **15,** displayed similar potency, with their IC_50_ equal to 0.036 and 0.057 µM, respectively. The selectivity of those structures towards desired mutated kinase was on par with dabrafenib. This, however, only partially transferred to the results of the cytotoxicity assay against BRAF^V600E^ mutated WM266.4 melanoma cells and BRAF^WT^ SK-mel-23 melanoma cells. Surprisingly enough dabrafenib did not showcase any selectivity towards mutated cells with SI = 0.55. While compound **13a** was not the most active one, with an IC_50_ equal to 4.52 µM, its selectivity index was notably more promising (SI = 11.1). Similar selectivity indexes showcased the most active compounds **11c** and **17** with an IC_50_ against WM266.4 equal to 1.53 and 1.24 µM respectively. It is worth noting that their effects in a previous analysis were also quite promising, with IC_50_ values for BRAF^V600E^ equal to 0.095 and 0.068 µM, respectively. On the other hand, compounds **7b** and **15** did not exhibit the expected selectivity against the tested cells. The article displayed promising results for thiazoles against BRAF^V600E^ mutated kinase. However, authors did not evaluate the newly synthesized compounds’ effects against normal cells nor did they assess the ADMET properties of their structures. Thus, further research into the compounds’ pharmacokinetic, pharmacodynamic and safety profiles should be performed in future works.

### 3.3. Tubulin Inhibition

**Figure 4:**
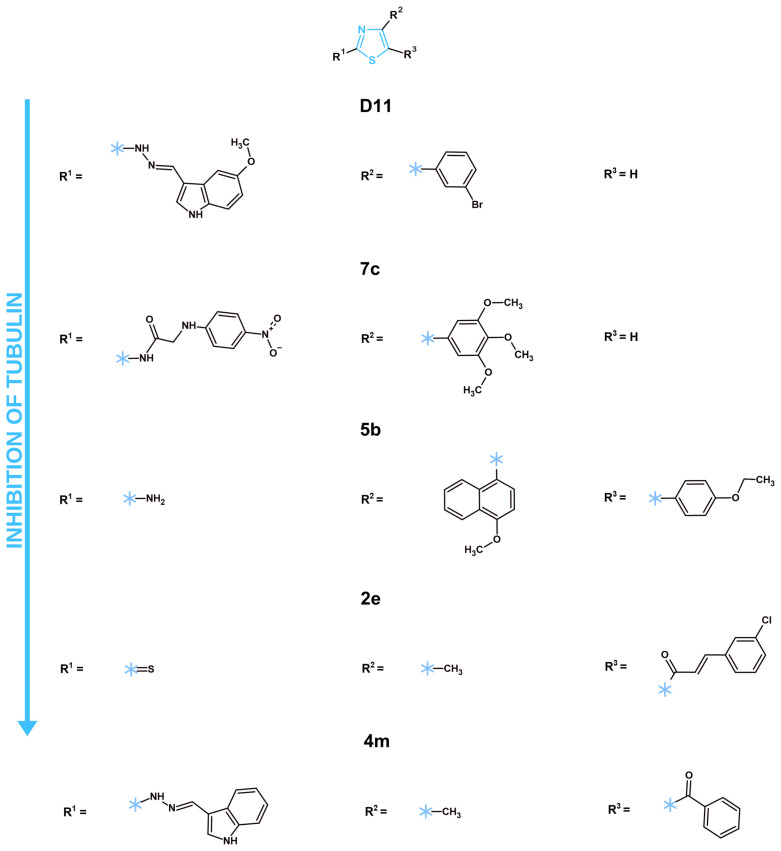
The structures of thiazole derivatives targeting tubulin inhibition published in 2021–2025. The compound names are preserved from their source publications for consistency.

Interestingly enough, among the most popular molecular targets for thiazole derivatives in recent years was tubulin [35,36,37,38,39] (Table 6; Figure 4). This protein consists of α- and β-tubulin parts that are connected together to form microtubules, which form the mitotic spindle. It takes a crucial part in cell division as it separates the chromosomes into two cells. In the majority of normal human cells, this process does not happen as often as in cancer cells. As such, targeting tubulin will affect cancer cells to a greater degree than normal cells. There are a number of already existing anticancer drugs targeting it, which can be divided in two main categories: tubulin polymerizing or microtubule stabilizing (Paclitaxel) and depolymerizing or microtubule disrupting (Vinca alkaloids, Colchicine) agents [105].

Some structural preferences for optimal tubulin-inhibiting activity of new promising structures were observed in the most prominent structures from each analyzed article. For instance, the most potent tubulin polymerization inhibitor, **D11**, showcased a 5-metoxy-indole moiety connected to the C2 position of the thiazole core via a hydrazone bridge. This may be contributed to the fact that indole is vastly present in established tubulin inhibitors, such as vincristine or vinblastine. Alternatively, potent tubulin polymerization inhibitor **7c** exhibited a p-nitrophenyl connected via a glycinamide bridge at the C2 position. The less active compounds **5b** and **2e** had simple amine group or sulfur at this position of their thiazole cores respectively. Such data may suggest that flexible chains with known effective moieties (**D11**) or polar functional groups (**7c**) are more promising structural substituents for the C2 position of the thiazole core. The C4 position also showcased a significant impact on the tubulin-inhibiting properties of thiazole derivatives. Directly connected substituted phenyl rings (**D11**, **7c**) or substituted 4-metoxy-naphthalene ring (**5b**) showcased more positive effects towards desirable outcomes in comparison to the simple methyl group (**2e**). However, it should be noted that **7c** has a trimethoxyphenyl moiety, which is a crucial part of the combretastatin A4 structure, one of the most potent tubulin depolymerizing agents. Most likely, this moiety is the main driver of the activity of compound **7c**. More interesting was the **5b** compound featuring 4-metoxy-naphthalene at the C4 position of its thiazole core. The methoxy group at the C4 position of the naphthalene ring is an electron-donating group. This provides an increase in the electron density of the naphthalene ring, which can strengthen π-π stacking interactions with aromatic amino acids in the tubulin binding pocket. Thus, despite a simple amine group at the C2 position of the thiazole core, **5b** remained an active tubulin polymerization inhibitor. Introducing substituents at the C5 position of the thiazole core caused a decrease in activity, as the most active tubulin polymerization inhibitors did not have any substituents at this position. While the **4m** compound did not have a quantified measurement of its tubulin polymerizing capabilities, its structure shows promise. The substituent at the C2 position of its thiazole core was nearly identical to the **D11** indole connected via hydrazone bridge. However, the methyl group at the C4 position and substituted C5 positions will cause a reduction in its activity.

In 2021, Wang et al. presented a series of thiazole–naphthalene derivatives with tubulin-inhibiting properties [35]. The authors explored the cytotoxicity of their compounds against MCF-7 and A-549 cell lines and identified the most active compound as **5b** with IC_50_ values equal to 0.48 and 0.97 µM, respectively. The authors analyzed this structure’s effects on tubulin polymerization in comparison to colchicine and obtained promising results. Compound **5b** displayed a similar tendency to reference compound to inhibit tubulin polymerization. Moreover, its IC_50_ for this analysis was equal to 3.30 µM, which was nearly three times lower in comparison to colchicine (9.10 µM). The compound also displayed a noticeable arrest in the G2/M phase of the cell cycle and promoted apoptosis in MCF-7 cells. The safety of the **5b** compound was confirmed against normal human HEK-293 cells, with IC_50_ = 16.37 µM, a notable difference in comparison to tested cell lines. However, a lack of pharmacokinetic and pharmacodynamic analysis was observed. Thus, an ample confirmation of the compound’s favorable ADMET characteristics is required before further advancement in drug development can be performed. In another article by El-Abd et al., the authors designed novel thiazole derivatives with a trimethoxyphenyl moiety [36]. The new compounds were tested for their cytotoxicity towards MCF-7, HepG2, HCT-116 and HeLa cancer cell lines. Other methods of anticancer research, including in vitro tubulin polymerization assessment and molecular docking, were performed by the authors. While compound **8** showcased the lowest IC_50_ in the cytotoxicity assay, compound **7c** exhibited superior effects as a tubulin depolymerizing agent in both the molecular docking (−14.15 kcal/mol) and in vitro tubulin polymerization enzymatic assay (IC_50_ = 2.00 µM), suppressing even the reference compound—combretastatin A4 (IC_50_ = 2.96 µM). Certain limitations of this work should be noted. A lack of ADMET properties assessment and analysis of cytotoxic activity against normal cell lines was observed. Without those methods of research, the pharmacokinetic, pharmacodynamic and safety profiles of the **7c** compound remain unknown and should be actively researched in the future. Another article featuring thiazole derivatives with tubulin-inhibiting capabilities was published by Wang et al. in 2023 [37]. Among the 26 novel structures presented by the authors, **D11** was identified as the most active compound due to its lowest IC_50_ in the tubulin polymerization assay (1.68 µM) and significant cytotoxic activity against MCF-7, A-549 and HeLa cancer cells with IC_50_ equal to 0.46, 0.21 and 0.32 µM respectively. A cell cycle analysis on A-549 cells further confirmed the dose-dependent tubulin-inhibiting effects of **D11**, as this structure arrested the cell cycle in the G2/M phase, a common occurrence for tubulin inhibitors, up to 39.58%, which was nearly double the amount of control with only 19.65% of cell population in G2/M phase. The authors also displayed the proapoptotic activity of the **D11** with 39.07% of the total apoptotic population at the highest tested concentration (1 µM). Finally, the authors visibly displayed dose-dependent tubulin-inhibiting effects on A-549 cells after 24 h of treatment via confocal microscopy, which showcased the rounding of the cells, microtubules concentrating around the cell nucleus, and, at the compound’s highest concentration, significant loss of microtubules with visible diffuse stain. Those characteristics are expected in tubulin depolymerizing agents such as combretastatin A4. While compound **D11** is undoubtedly a confirmed potent tubulin inhibitor, its safety towards normal human cell lines and ADMET properties remain unknown as it was not tested by the authors. As of right now, this compound will require further research into its safety, pharmacodynamic and pharmacokinetic profiles in future works. Another work focusing on thiazole derivatives as promising tubulin inhibitors was published by Aggarwal et al. in 2024 [38]. In this work, the authors presented a series of arylidenehydrazinyl-thiazoles, which were tested against breast cancer (MCF-7, BT-474), lung cancer (A-549), leukemia (MOLT-4), and pancreatic cancer (BxPC-3) cell lines. Firstly, the authors examined all compounds at 10 µM concentration to identify the most promising structures, which were **4m, 4n** and **4r**. Next, the authors analyzed the cytotoxicity of those compounds in a dose-dependent manner against all the above-mentioned cell lines. While **4m, 4n** and **4r** were tested in the tubulin polymerization assay and displayed inhibiting capabilities comparable to colchicine, the IC_50_ or the maximal velocity were not mentioned in the manuscript. It is worth mentioning that the cell cycle analysis also exhibited a significant cell cycle arrest in MOLT-4 cells in the G2/M phase. On the other hand, the authors also analyzed the most active compounds’ capabilities via the intrinsic pathway, which was confirmed by increased caspase 3/7 and notably decreased mitochondrial potential. Taking into consideration the lack of an ADMET properties analysis, studies against normal cell lines and insufficient quantitative data regarding tubulin inhibition caused by synthesized structures, further research is required for those compounds. Lastly, Hashem et al. published an article featuring thiazole–chalcone derivatives (**2a–2p**) with tubulin as its main molecular target [39]. The NCI-60 protocol was applied to those compounds to identify the ones with the most significant potential. As a result of that research, compounds **2e**, **2g**, **2h** and **2p** were chosen for the tubulin polymerization assay. The closest to the reference drug, combretastatin A4 (IC_50_ = 4.93 µM), was structure **2e**, which exhibited an IC_50_ equal to 7.78 µM. Other in vitro analyses were not displayed in this manuscript. The ADMET profiles of the new compounds were assessed utilizing in vitro approach. The new structures satisfied Lipinski rules, did not penetrate the BBB, had favorable bioavailability and exhibited promising GIT absorption. Nonetheless, compound safety analysis and a more detailed mechanistic assessment of compound cytotoxic activity is required before proceeding to further steps in drug development.

### 3.4. Other Targets and Mechanisms of Anticancer Activity Aimed by Thiazole Derivatives in 2021–2025

**Figure 5:**
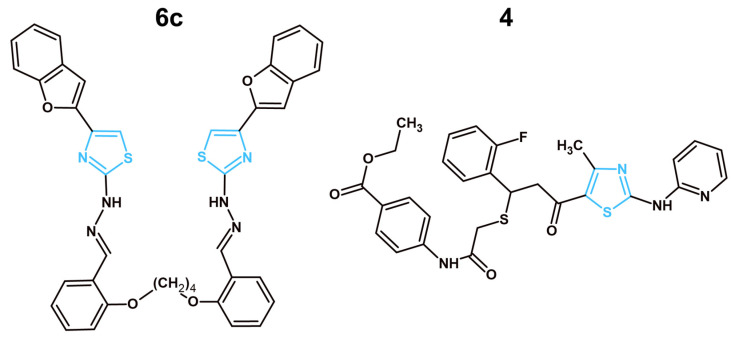
The structures of thiazole derivatives targeting PI3K (**6c**) and PARP-1 (**4**) published in 2021–2025. The compound names are preserved from their source publications for consistency.

PI3K and PARP-1 are widely known targets in anticancer research, which, however, were not studied extensively in recent years for thiazole derivatives. An important role of the PI3K/AKT pathway in EGFR and VEGFR-2 inhibition for cell survival was already mentioned previously in this manuscript. Alternatively, this protein plays a crucial part in the regulation of cell growth and dividing. After being activated, PI3K converts PIP_2_ (Phosphatidylinositol 4,5-bisphosphate) to PIP_3_ (Phosphatidylinositol (3,4,5)-trisphosphate), which plays a part as a docking site for AKT. Activating AKT increases stimulation in mTOR, which leads to increased protein synthesis and ribosome production, preparing cells for dividing. A mutation in PI3K will cause the cell to uncontrollably divide, leading to cancer. Furthermore, the PI3K/AKT pathway affects cytoskeletal dynamics, promoting actin polymerization, cell migration, and stability of microtubules [106]. For example, Radwan et al. presented a series of novel bis-thiazoles with benzofuran (**6a–f**) or benzothiazole (**7a–f**) moieties developed through a molecular hybridization approach with PI3K as the main molecular target in mind (Figure 5) [50]. The new compounds were tested in vitro against MCF-7, HCT-116, and HepG2 cancer cell lines. The obtained data showcased that derivatives **6c** and **7e** were the most active among the tested, with an IC_50_ on MCF-7 cells equal to 9.49 and 7.31 µM respectively. Next, those two compounds were chosen for the in vitro enzymatic activity assay with PI3Kα as the target. Compound **6c** showcased a significantly more impressive result in this study with an IC_50_ equal to 9.6 × 10^−5^ µM, which was comparable to the reference drug alpelisib (IC_50_ = 6.79 × 10^−5^ µM) and considerably better than **7e** (IC_50_ = 2.8 × 10^−4^ µM). Compound **6c** was further analyzed for its effects on cell cycle and apoptosis induction in MCF-7 cells. The most active structure displayed a cell cycle arrest in S phase with 52.19% of cell population in that phase, which was notably higher than in the untreated control (34.75%). Furthermore, compound **6c** displayed a significant increase in the apoptotic population of MCF-7 cells, with 38.25% total apoptotic cells in comparison to the control with only 1.86%. The proapoptotic effects of **6c** were further confirmed by Western blot analysis, showcasing an upregulating trend in one of the key proapoptotic proteins—p53 protein. Nonetheless, several limitations of this article should be noted. The authors did not perform any cytotoxicity assessments against normal cells and no ADMET properties were analyzed. Without such crucial information, further development for clinical application cannot be performed. Compound **6c** requires proper analysis of its pharmacokinetic, pharmacodynamic and safety profiles in future studies.

The anticancer activity of PARP-1 novel inhibitors with a thiazole core was researched by Ivasechko et al. (Figure 5) [53]. PARP-1, also known as poly-ADP-ribose polymerase 1, is an enzyme that makes a significant contribution to the DNA repair mechanisms of the cell. If blocked, DNA damage will continuously accumulate, leading to cell death. In the case of normal cells, even with PARP-1 inhibition, DNA damage can still be repaired with other mechanisms, such as homologous recombination. This, however, is not the case for various types of cancer due to mutations preventing those mechanisms. As such, targeting PARP provides certain selectivity towards cancer cells [107]. In the manuscript, the authors performed an NCI-60 protocol screening and an MTT cytotoxicity assay to identify the most active structure. Compound **4** was identified as such due to its strong effects across numerous tested cell lines (HCT-116, MCF-7, Jurkat, A-549, SK-OV-3, KB3-1) and safety towards the majority of normal human cells (HEK-293 and HaCaT). MCF-7 cells were the most prone to compound **4** activity, with an IC_50_ equal to 2.79 µM after 72 h of treatment. Compound **4** displayed significant proapoptotic ability by increasing the total apoptotic cell population in MCF-7 breast cancer cells up to 42.7%, a major difference from the control with only 4.4%. This structure also managed to notably decrease the mitochondrial potential of the MCF-7 cells, suggesting that compound **4** can activate apoptosis via the intrinsic pathway. To showcase its effects on PARP-1 protein, the authors preincubated MCF-7 cells with a low concentration of the verified PARP-1 inhibitor, Fluzoparib. Afterwards, the cells were treated with compound **4** and compared to cells that were treated with only compound **4** without Fluzoparib. The obtained data showcased a statistically significant decrease in cytotoxicity in the pretreated cells, suggesting that both compound **4** and Fluzoparib are targeting the same molecular target. Moreover, the authors also tested the compound **4** with other inhibitors of DNA-repairing proteins (Lomeguatrib, Bractoppin). The analyzed results showcased an increase in the cytotoxicity of compound **4** in combination with Lomeguatrib up to three times, which was not observed in the case of Bractoppin. This provides an interesting insight into combination therapy of various inhibitors of DNA-repairing proteins. Overall, the authors provided some evidence towards the proapoptotic effects of compound **4** and its PARP-1-inhibiting capabilities. Taking into consideration the lack of ADMET assessment and insufficient mechanistic studies towards PARP-1, further research is required.

**Figure 6:**
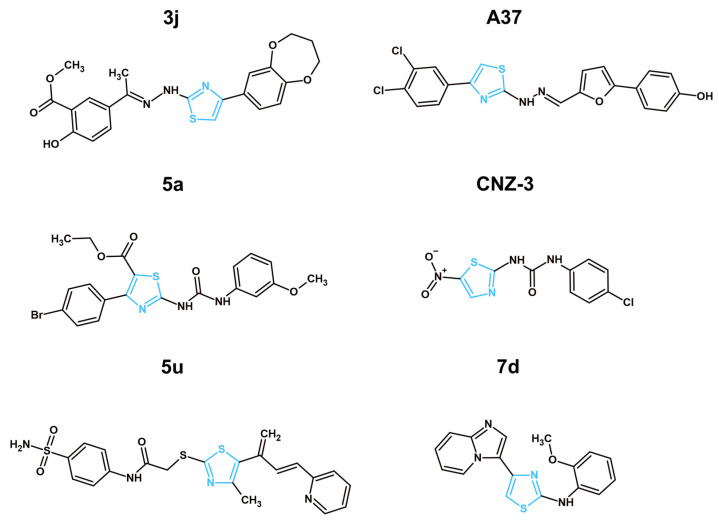
The structures of thiazole derivatives targeting PTP1B (**3j**), eIF4E (**A37**), SIRT2 (**5a; CNZ-3**), CA IX and XII (**5u**) and PKM2 (**7d**) published in 2021–2025. The compound names are preserved from their source publications for consistency.

Among less widely known molecular targets for anticancer research of novel thiazole derivatives in recent years were PTP1B [54], SIRT2 [51,52], CA IX and XII [55], PKM2 [56], and eIF4E (Figure 6) [57]. A compelling reasoning behind targeting PTP1B was proposed by Kołodziej-Sobczak et al. in their paper [54]. As written by the authors, protein tyrosine phosphatases are very similar in their function to protein tyrosine kinases (PTKs). While PTKs are responsible for adding a phosphatase group to the proteins, PTPs are working by removing those phosphatases. Despite this, there are no FDA-approved anticancer compounds targeting protein tyrosine phosphatases. This information signifies the overlooking of PTPs as potential molecular targets for anticancer research [108,109]. Moreover, PTP1B overexpression is present in breast cancer and can have negative effects in terms of cancer progression. For those reasons, Kołodziej-Sobczak et al. synthesized 14 new thiazole derivatives with methyl salicylate groups, due to the fact that methyl salicylate was present in the previously described PTP1B inhibitor. Firstly, the compounds were tested for their capability to inhibit the expected molecular target. Compounds **3j**, **3e** and **3d** showcased the most promising results with IC_50_ ranging from 0.93 to 0.51 µM. Afterwards, those three compounds were analyzed for their cytotoxicity against breast cancer T47D, lung cancer A-549 and the normal human fibroblast HSF cell line. Compound **3j** was identified as the most active structure due to its high selectivity index (10.90) against T47D in comparison to HSF. The IC_50_ for **3j** against T47D was equal to 21.21 µM, a significant difference from 231.3 µM against fibroblast cells. Compound **3j** was also analyzed for its effects on the cell cycle in T47D cells. The obtained results displayed that **3j** significantly increased the population of the S phase of the cell cycle in T47D cells at 20 and 40 µM concentrations. The selected structure also displayed proapoptotic effects by inducing apoptosis up to 23.21%, a noticeable difference in comparison to the untreated control with only 5.87% of apoptotic cells. Despite the promising selectivity and safety, a lack of ADMET properties assessment was observed. Thus, a detailed analysis of the pharmacokinetic and pharmacodynamic properties of **3j** is required.

In their work, Piacente et al. actively researched novel thiazole-bearing structures targeting SIRT2 [51]. This deacetylase plays an important part in numerous biological processes of the cell including cell cycle and mitosis regulation, oxidative stress and autophagy, neurodevelopment and neurodegeneration, genome integrity and metabolism. However, its unique nature is in its ability to be both tumor suppressor or oncogene depending on cancer type, stage of the disease, and/or active pathways in the tumor. As such, both activators and inhibitors are being researched in terms of their anticancer properties [110]. Utilizing in vitro drug design and a ligand-based approach, Piacente et al. created a series of new potential SIRT2 inhibitors. Next, the authors tested their structures for the IC_50_ concentration required for SIRT2 inhibition. Compound **5a** was found to be the most active structure with an IC_50_ equal to 9 µM. To analyze the novel compounds’ selectivity, the authors also researched them for their inhibition of SIRT-1, 3, and 6. It is worth noting that the new structures were selective towards SIRT1 and SIRT2, while SIRT3 and SIRT6 were relatively unaffected by them. Compound **5a** was further tested for its cytotoxicity against skin cancer SCC13 and human keratinocytes HaCaT cell lines. The gathered results displayed that the **5a** compound was more active against cancer cells rather than normal human cells. Moreover, to confirm the compound’s effects on SIRT2, the authors utilized a Western blot research method. They tested the compound’s capability to increase the acetylation of α-tubulin, one of the main SIRT2 substrates. Compound **5a** exhibited a visible increase in relative protein expression of acetylated α-tubulin, thus confirming its SIRT2-inhibiting effects. While the findings of the authors are promising, it would be beneficial to test more cell lines in future research, as well as conduct a more in-depth analysis of the compound’s effects on mechanisms connected to SIRT2 processes such as apoptosis, autophagy and cell cycle. Moreover, an ADMET analysis for **5a** should be performed in the future as well, since it was absent in the analyzed manuscript. Another article featuring an SIRT-2-inhibiting compound with a thiazole ring was published by Vázquez-Bautista et al. in 2025 [52]. The authors analyzed a number of new structures mainly featuring a benzimidazole fragment, except one compound, which had a thiazole ring as its core (**CNZ-3**). It should be noted that this structure exhibited the highest overall anticancer activity against tested glioblastoma cell lines A172 and U87-MG. Unique to this compound was its particularly stronger effect under hypoxia conditions in comparison to normoxia. For instance its IC_50_ against A172 was equal to 12 and 9 µM under normoxia and hypoxia conditions, respectively. Comparatively, the benzimidazole derivatives had at least three times higher IC_50_ values under both conditions. Moreover, unlike other analyzed structures, **CNZ-3** was consistent in its significant downregulating activity against key targeted proteins SIRT2, VEGF and G6PD (Glucose-6-Phosphate Dehydrogenase) under hypoxia. According to the authors, hypoxia in glioblastoma cell lines can often lead to increased drug resistance. As such, the results obtained regarding **CNZ-3** are especially promising. However, it should be noted that the **CNZ-3** structure contains an active nitro group at the C5 position of the thiazole core. According to the authors, this group is highly responsible for the activity against targeted proteins. Nonetheless, the authors also acknowledged potential risks in terms of the possible undesirable toxic effects of the present nitro group. Additionally, no ADMET assessment was performed for the new structures and no testing against normal cells was done. Further research in terms of the compounds’ pharmacokinetic, pharmacodynamic and safety profiles should be performed in the future. Another article featuring novel thiazole-containing compounds targeting the hypoxia-related adapting mechanism was published by Elshamsy et al. [55]. The authors targeted carbonic anhydrases IX and XII, since those enzymes are actively overexpressed in cancer, especially under hypoxic conditions. In fact, those two enzymes are induced under limited oxygen conditions. The main function of those enzymes is pH regulation. CA IX and XII maintain acid–base balance by catalyzing the carbon dioxide into bicarbonate and protons. Thus, in the lack of oxygen, the cancer cells, heavily reliant on glycolysis, induce expression of those enzymes to combat the carbon dioxide and lower pH, allowing them to continue to proliferate [111,112]. In their article, Elshamsy et al. synthesized new structures with a thiazole ring based on the promising SLC-0111 compound, which is currently in clinical trials as an anticancer agent targeting CA IX. The authors applied a tail-focused design incorporating a thioacetamide linker instead of the urea linker present in SLC-0111. This linker fragment connected the thiazole ring at the C2 position to the crucial-for-activity phenyl sulfonamide moiety. Alternatively, the C5 position of the thiazole ring had a fragment of chalcone-based substituents. The most promising structure, compound **5u**, had substituted the phenyl ring of chalcone fragment with heterocyclic 2-pyridinyl. **5u** showcased potent inhibition constants for CA IX and XII equal to 15.5 and 2.0 nM, respectively. This result outperformed the reference compound, which had only 45.0 and 4.5 nM for CA IX and XII, respectively. However, the **5u** compound exhibited significantly less selectivity effect for the targeted enzyme. Its off-target inhibition constants for CA I and II were equal to 159.2 and 4.8 nM respectively, a significant difference in comparison to SLC-0111 with 5080.0 and 960.0 nM, respectively. Nonetheless, **5u** displayed potent anticancer activity against a panel of NCI-60 cancer with IC_50_ values against most cell lines less than 2.0 µM. Especially should be noted its performance against melanoma cell lines A375 and A2058 under hypoxic conditions. IC_50_ values after 72 h of treatment against those two cell lines were equal to 3.2 and 3.7 µM respectively, significantly outperforming SLC-0111 with 106.7 and 104.9 µM, respectively. Regarding the safety of the novel compound, **5u** showcased an IC_50_ over 100 µM against the fibroblast L929 cell line. ADMET properties were assessed via in vitro approach. According to the authors, **5u** did not violate any of the Lipinski rules, providing good potential for the bioavailability of this compound. Combining with the high IC_50_ concentration against normal cell line, this compound has a promising ADMET profile. However, certain concerns should be noted. The selectivity of **5u** towards the molecular target was significantly lower than the reference compound. The lack of mechanistic studies into the effects of the new structures on the mechanism of cell death and overall impact on the targeted cell lines should be noted. Thus, **5u** requires more extensive analysis through mechanistic studies of the proposed mechanism of anticancer activity. Das et al. showcased their newly synthesized thiazole-bearing molecules targeting PKM2 [56]. According to the authors, this enzyme has a significant influence on the process of glycolysis. Its overexpression, present in triple-negative breast cancer, can enhance glucose uptake and lactate production, providing necessary nutrients for tumor growth. Aiming for PKM2, the authors wanted to starve the tumor. All newly synthesized thiazole derivatives were tested for their cytotoxic activity against COLO-205, A549, 4T1 and MCF-7 cell lines and their capability to inhibit PKM2. Among the novel structures, **7d** was identified as the most active compound due to its low IC_50_ concentration required for PKM2 inhibition (0.023 µM) and cytotoxicity across chosen cell lines, especially MCF-7 (IC_50_ = 19.80 µM) and 4T1 rodent breast cancer cell line (IC_50_ = 14.38 µM). Furthermore, compound **7d** exhibited a notable safety profile, showcasing cell viability equal to 92.92% in normal human breast cells MCF-10A. The chosen structure displayed promising results in other in vitro analyses. **7d** arrested the cell cycle in the G2/M phase, promoted apoptosis in 4T1 cells in both 2D and 3D cultures and significantly reduced mRNA expressions of PKM1 and PKM2. Lastly, the authors performed in an vivo analysis for the **7d** compound in a 4T1 mouse model over a 21-day treatment period. The obtained data was very promising as **7d** managed to not only cause significant tumor regression but also allowed the mouse to regain body weight during the treatment time. A biomarker analysis exhibited a noteworthy reduction in PKM2 expression in tumor tissues and a reduction in VEGF expression, suggesting the prevention of the formation of new blood vessels, essentially starving the tumor. The overall results are notably optimistic as the authors showed the expected effects in both in vitro and in vivo models, providing crucial information that can be utilized for further research in the future. However, a lack of ADMET assessment was noted. A detailed analysis into **7d**’s pharmacodynamic and pharmacokinetic profile should be performed in the future. In the article published by Yao et al., the authors showcased novel thiazole derivatives connected to furan via hydrazinyl targeting the MAPK signaling pathway through binding to eIF4E [57]. Both thiazole and furan rings had phenyl rings connected to them with different substitutes. After performing a cytotoxicity analysis on HepG2, A-549, HeLa (melanoma), MCF-7 cancer cells and normal non-cancerous human embryonic kidney epithelial cells (HEK-293T), the most active compound, **A37,** with 4-hydroxyphenyl connected to the furan ring and 3,4-dichlorophenyl connected to the thiazole ring, was identified. This structure showcased an IC_50_ below 5 µM in all tested cancer cell lines, while normal cells were not affected in the highest tested concentration (40 µM). For those reasons, this compound was chosen for more in-depth research. Surface plasmon reference (SPR) analysis was utilized to analyze the **A37** compound’s capability to bind to eIF4E. The obtained data showcased a dissociation constant (K_D_) equal to 20.2 µM, which was slightly better than the reference compound 4EGI-1 with dissociation constant equal to 25 µM. Next, the Western blot method was applied to display how this binding to eIF4E would affect the MAPK signaling pathway. The performed analysis confirmed a notable reduction in the relative protein expressions of Ras, p-MNK, p-ERK and p-eIF4E in HepG2 cells. **A37** also exhibited proapoptotic effects in a dose-dependent manner, where the total apoptotic cell population in HepG2 cells was equal to 49% and 74% for 2.5 and 5 µM, respectively. Furthermore, **A37** also increased relative protein expression of main pro-apoptotic proteins Bax and Caspase-3 and decreased the levels of anti-apoptotic Bcl-2 protein. Most importantly, the authors performed in vivo research in mice HepG2 xenografts. **A37** reduced the tumor growth by 36% at 25 mg/kg and by 68% in 50 mg/kg on the 18th day. Especially promising was the fact that the mice did not display a notable loss of body weight or significant morphological damage to other organs. While the authors performed extensive work to showcase that their potent, safe, and selective **A37** compound can become a potential anticancer drug candidate, research into the ADMET properties of this structure should be performed in the future before proceeding to next steps in drug development.

## 4. Mechanisms of Anticancer Activity of Novel Derivatives with 4-Thiazolidinone/Thiazolidinedione Ring in 2021–2025

### 4.1. Targeting Tyrosine Kinases

#### 4.1.1. VEGFR-2 Inhibition

**Figure 7:**
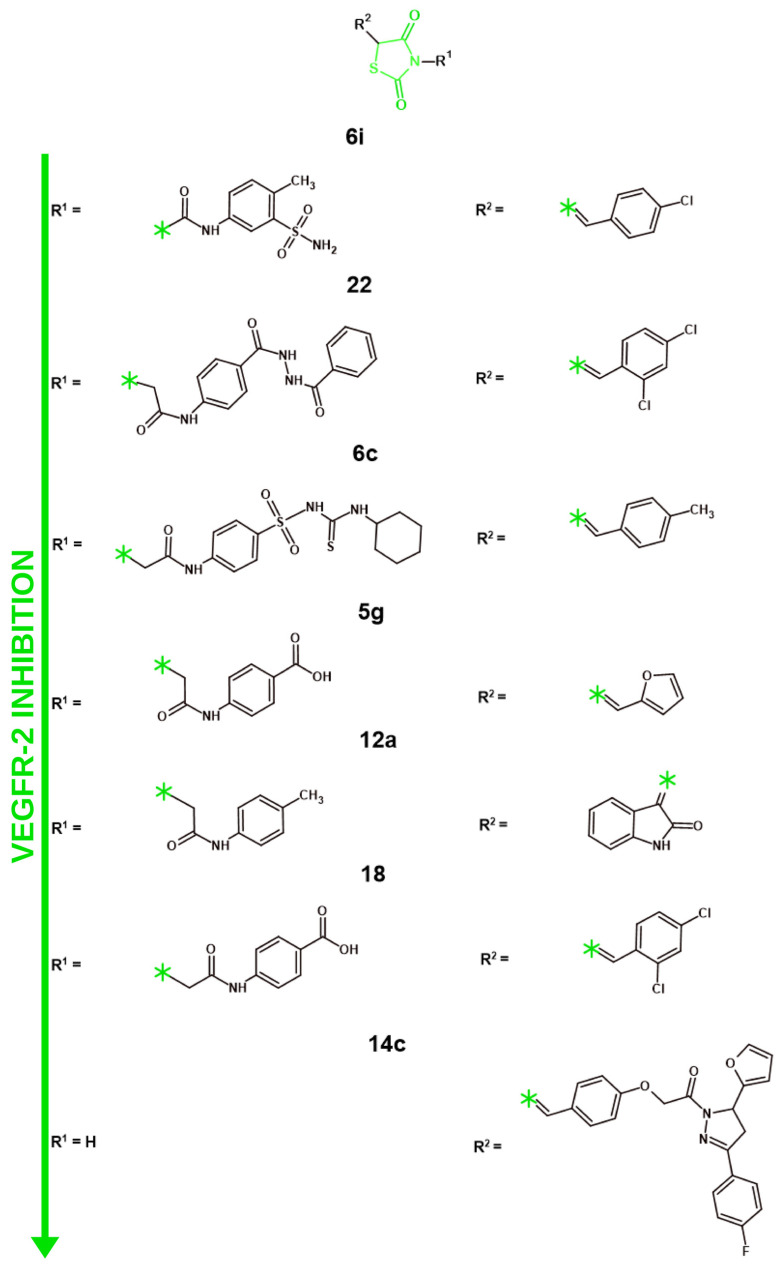
The structures of 4-TZD/TZD derivatives targeting VEGFR-2 inhibition published in 2021–2025. The compound names are preserved from their source publications for consistency.

Similarly to 4-thiazole derivatives, among the most popular molecular targets in recent years for anticancer research was VEGFR-2 (Figure 7; Table 7). Seven studies reported new TZD derivatives targeting VEGFR-2 in recent years. It is worth noting that a certain degree of structural similarity was observed between the most promising compounds of most of the analyzed manuscripts. Acetamide linked phenyl ring with various derivatives was observed at the N3 position of the core structure for six out of seven structures, including the most active compound **6i**. However, the least active VEGFR-2 inhibitor, **14c**, did not have a substitute at the N3 position, providing crucial information for the necessity of such a substitute for potent VEGFR-2 inhibition. Regarding substitutes at the benzene ring, the para position was the most popular place. While potent structures such as **22** and **6c** exhibited bulkier fragments at the para position, the most active compound, **6i**, showcased a smaller methyl fragment, similarly to the less active **12a** TZD derivative. The important difference, however, was the sulfonamide present at the meta position of the benzene ring. While sulfonamide was also present as a part of the para substitute of **6c**, its amine was further substituted. Such information might suggest the need for the primary sulfonamide for optimal VEGFR-2 inhibition. Another important part was the substitutes at the C5 position. The least active structure, **14c**, showcased a bulky fragment with multiple phenyl rings and five-membered heterocyclic systems. Comparatively, more active compounds exhibited more simple structures featuring one heterocyclic system with simple substitutes. In contrast, the most potent compounds featured smaller aryl or heterocyclic systems, such as the 4-chlorobenzylidene fragment in **6i,** 2,4-dichlorobenzylidene in **22**, 2-methylbenzylidene in **6c**, or the furan and oxindole systems in **5g** and **12a**. These findings confirm that a compact, aromatic system at C5 is necessary for effective VEGFR-2 binding.

However, tested VEGFR-2-inhibiting capabilities do not necessary translate to potent anticancer activity, since a significant number of factors, such as bioavailability and selectivity, can affect compounds’ capability to affect cancer cells. Upadhyay et al. presented a series of novel diarylpyrazoline thiazolidinedione derivatives with a dual-targeting mechanism of activity against VEGFR-2 and HDAC [58]. While the effects of VEGFR-2 inhibition were previously described in this manuscript for thiazole derivatives, the inhibition of HDAC is a mechanism of activity that was not explored for thiazole derivatives in recent years. Those enzymes perform the deacetylation of histones, which consolidate their connection to DNA, resulting in a closed chromatin structure, essentially decreasing gene transcription. On the other hand, HDACs also affect a great number of various proteins that are directly affecting the most important processes for anticancer research, including apoptosis and cell cycle, cell differentiation and immune response. The functionality of p53, STAT3, NF-kB (nuclear factor kappa B), Myc, β-catenin, HIF-1α (Hypoxia-Inducible Factor-1 alpha) and RUNX3 (runt-domain transcription factor) is regulated by HDACs. As such, a number of HDAC inhibitors have been FDA-approved for anticancer therapy: Vorinostat, Romidepsin, Belinostat, Panobinostat and Trametinib [113,114]. Among the newly synthesized compounds by Upadhyay et al., compound **14c** was identified as the most promising structure due to its selective inhibition of HDAC4 (IC_50_ = 0.88 µM), since this compound did not significantly affect other HDAC-1, 2, 3, 7, and 8 proteins. The **14c** compound displayed the most notable effect on VEGFR-2 inhibition among the new structures as well, with an IC_50_ equal to 5 µM, which, however, was ten times worse than that of the reference drug—staurosporine (IC_50_ = 0.5 µM). To confirm the anti-angiogenic activity of **14c**, HUVEC proliferation was analyzed. The IC_50_ of **14c** against HUVEC was similar to the reference drug—staurosporine, with IC_50_ equaling to 0.7 and 0.5 µM, respectively. This, however, did not correlate with the cell migration and capillary-like tube formation assays on HUVEC cells, whereas the **14c** compound displayed a slight effect in comparison to the reference drug. On the other hand, in a CAM assay, the **14c** compound visibly reduced the number of branching capillaries in comparison to the untreated control. The cytotoxicity of **14c** was tested against a number of cancer cell lines (HT-29, A-549, MCF-7, K-562), with A-549 and HT-29 being the most prone to the activity of the **14c** compound, with IC_50_ values equal to 19.52 µM and 18.84 µM, respectively. While this article provides a number of unique methods of analyses such as capillary-like tube formation or CAM assays, the results were not very consistent throughout all of the anticancer methods of analysis. Moreover, the ADMET properties and safety profile of the novel structures were not explored, nor were the effects of **14c** on cell cycle or apoptosis. As such, further extensive research into these compounds should be performed in the future. In another article by Taghour et al., the authors explored a molecular hybridization approach to synthesize novel thiazolidinedione derivatives featuring structural fragments from FDA-approved drugs that can inhibit VEGFR-2 [59]. Compounds **12a, b** were based on Sunitinib with an oxindole fragment, while compounds **8a–c** were premised on Cabozanitinib with the addition of 2-oxo-1,2-dihydroquinoline to the 4-thiazolidinone pharmacophore. The novel structures’ cytotoxic effects were analyzed against Caco-2 (colon cancer), HepG2, and MDA-MB-231 cancer cell lines and Vero, monkey normal kidney epithelial cells. Among the new structures, **12a** was identified as the most active compound due to its superior cytotoxicity among the tested cell lines. The most affected cell line was Caco-2, with the IC_50_ equal to 2.00 µM for **12a**. This structure also displayed an astonishing safety profile with SI^Vero/Caco−2^ = 365. The inhibiting properties towards VEGFR-2 of novel structures were measured utilizing an enzyme inhibition assay. The obtained data provided by the authors showcases that, while compound **12a** was the most cytotoxic against cancer, its IC_50_ required for VEGFR-2 inhibition (0.116 µM) was less than that of **12b** or **8a, b,** whose IC_50_ values ranged from 0.084 to 0.097 µM. However, taking into consideration the cytotoxicity results of the new structures, compound **12a** was still chosen for further analysis. **12a** displayed a notable inhibition of cell migration after 24 h of treatment against Caco-2 cells. The proapoptotic effects of **12a** were analyzed with the use of RT-qPCR. **12a** downregulated the expressions of numerous anti-apoptotic proteins, namely Bcl-2 and Survivin. On the other hand, **12a** showcased significant upregulation of TGF, a tumor suppressing protein. Compound **12a** exhibited significant promise for further research, considering its anticancer effects and notable selectivity indices against normal cells. Furthermore, an in vitro analysis of ADME properties and potential toxicity revealed promising results. **12a** did not exhibit the capability to penetrate the BBB or cause carcinogenicity. Moreover, this compound also exhibited a higher oral LD_50_ than sorafenib or sunitinib. Alternatively, solubility was predicted to be low with good absorption level. Similarly to Sorafenib, compound **12a** might cause mild irritation to the eyes, with no predicted irritation to the skin. Such results create a promising ADMET profile for the main structure of the manuscript. In another article published by Abdelgawad et al., the authors explored novel thiazolidinediones with sulfonylthiourea structural fragments targeting two different activities, anticancer and anti-hyperglycemic [60]. The addition of the sulfonylthiourea fragment was done to extend the core structure and enhance the binding affinity towards VEGFR-2. Furthermore, this structural fragment should act as a sulfonylurea receptor agonist, which is particularly important for its anti-hyperglycemic activity, as sulfonylurea receptors are actively involved in insulin secretion. Surprisingly, two compounds featuring a cyclohexyl radical, **6c** and **7c,** were the most cytotoxic among the structures presented by the authors. Their activity on tested cancer cell lines (HCT-116, MCF-7, HepG2) were comparable to each other and ranged from 5.77 to 8.99 µM. Similarly to the cytotoxicity assay results, the VEGFR-2 inhibition assay also displayed corresponding data, with both compounds’ IC_50_ equal to 0.08 µM, a slightly better result in comparison to the reference compound—sorafenib (IC_50_ = 0.10 µM). A PPARγ assay showcased that both 6**c** and 7**c** exhibited similar results with their IC_50_ equal to 0.300 and 0.296 µM, nearly identical to the reference compound—rosiglitazone (IC_50_ = 0.292 µM). The EC_50_ of both **6c** and **7c** in the insulin secretion assay were identically equal to 0.70 µM. The compounds’ safety profile was explored by testing them against Vero cells. Interestingly enough, **6c** proved to be slightly more active against normal cells with an IC_50_ equal to 49.26 µM, while **7c** was less active with an IC_50_ equal to 68.25 µM. The ADMET properties of the synthesized structures were assessed utilizing in vitro approach. It is worth noting that compounds **6c** and **7c** mostly adhered to the Lipinski rules, violating only one rule. Promising GIT absorption rates were predicted for those structures. Worrying CNS penetrating capabilities were observed. Alternatively, the metabolic predictions for **6c** and **7c** were promising. In comparison to Sorafenib, those structures did not exhibit the capability to inhibit various cytochromes (CYP3A4, CYP2C9, CYP2C19, and CYP1A2) and were showcased to be a substrate only to CYP3A4. While the authors provided important data regarding the anticancer and anti-hyperglycemic activity of their novel structures, further in-depth research would be beneficial in the future. Especially so, considering that the connection between the PPARγ agonistic effects of **6c** and **7c** and their anticancer activity was not deeply explored. The compounds’ effects on apoptosis were not analyzed either. Another study featuring novel thiazolidinedione derivatives was published by Aziz et al. The authors explored the potential modifications of already existing drugs, VEGFR-2 inhibitors sorafenib and sunitinib, and EGFR inhibitor erlotinib, to synthesize novel dual-targeting agents [61]. The authors devised to replace the central aryl ring in those structures with a thiazolidinedione pharmacophore. Further changes in structure were made, mainly an introduction of either thiophene (**4a–g**) or furan (**5a–g**) fragments. The new structures were tested against four cancer cell lines: HepG2, MCF-7, A549 and HCT-116. Compound **5g** was the most promising among them, with its IC_50_ equal to 3.86 µM against HepG2 cells, a similar result to the reference structure—Sorafenib (IC_50_ = 4.00). **5g** also displayed the most notable effects on both VEGFR-2 inhibition and EGFR^T790M^ inhibition, with IC_50_ equal to 0.080 and 0.14 µM, respectively, which was comparable to or better than the reference compounds, with their IC_50_ equaling to 0.084 and 0.24 µM respectively. An in vitro approach was utilized to predict the ADMET properties of the novel structures. Structure **5g** did not violate any of the Lipinski rules; it showcased promising GIT absorption and exhibited no capability to affect any of the metabolic cytochromes. Alternatively, **5g** exhibited concerning CNS permeability and a potential predicted hepatotoxic effect, a result similar to Sorafenib and Erlotinib. The authors showcased promising results regarding the dual-targeting mechanism of the activity of their novel structures; nonetheless, a detailed analysis of the anticancer effects of those compounds and their in vitro and/or in vivo safety profiles were not explored. While the authors presented them as a template for future design, further in-depth research would be beneficial in the future. Novel thiazolidine-2,4-dione derivatives as potential VEGFR-2 inhibitors were also explored by Eissa et al. [62]. The new compounds’ cytotoxicity was analyzed against MCF-7 and HepG2 cancer cell lines and Vero normal cells and their effects on VEGFR-2 inhibition. Among the tested compounds, compound **22** showcased significantly more prominent effects on VEGFR-2 inhibition with an IC_50_ equal to 0.079 µM. Its anticancer activity against tested cell lines, while prominent, was not the most potent among novel thiazolidinedione derivatives, with an IC_50_ equal to 2.04 and 1.21 µM for HepG2 and MCF-7, respectively. However, taking into consideration its notably better results on VEGFR-2 inhibition, compound **22** was identified as the most promising structure. Next, compound **22**’s effects on the cell cycle of MCF-7 cells were analyzed. Compound **22** showcased a slight increase in the S phase of the cell cycle (33.84%) in comparison to untreated cells (24.61%). Moreover, structure **22** also notably increased the total apoptotic population up to 35.68%, a notable difference from untreated cells with only 0.44% apoptotic cells. Scratch assay results confirmed its inhibiting effects on cell migration, as the wound closed only by 30.20% after 48 h, in comparison to 81.62% in untreated control cells. The safety of compound **22** was explored by analyzing its cytotoxicity against normal Vero cells. The IC_50_ of the compound **22** was equal to 3.97 µM in this experiment. As such, with compound **22**’s SI^Vero/MCF−7^ equaling to 3.28, it was deemed mildly selective towards cancer cells. In vitro predictions showcased a favorable ADME profile, with compound **22** not violating Lipinski rules, exhibiting a very low level of BBB penetration and aqueous solubility better than that of Sorafenib. However, a concerningly low GIT absorption was observed. Alternatively, toxicity predictions provided promising results with compound **22** exhibiting a non-mutagenic and non-carcinogenic nature. The analyzed structure is predicted to cause only mild irritation to the eyes and no dermal irritation. The obtained data suggests that compound **22** has a promising predicted ADMET profile, mild selectivity towards cancer cells and significant impact on VEGFR-2 protein. However, a more detailed mechanistic analysis and in vitro and/or in vivo confirmation of pharmacokinetic and pharmacodynamic properties is required before further drug development. In another article presented by El-Adl et al., the authors designed novel thiazolidinedione derivatives targeting VEGFR-2 utilizing molecular hybridization and bioisosteric replacement strategies [63]. The aryl core of Sorafenib was changed to thiazolidinedione pharmacophore, while the hydrophobic tail of novel derivatives was either 4-chlorobenzylidene or 2,4-dichlorobenzylidene moieties. One of the main differences between compounds was in linkers with hydrogen bond acceptor/donor groups designed to interact with crucial amino acids. Compounds **4–6** featured an ester linker, while **7–19** had an amide linker. All the new structures were tested against MCF-7, HCT-116 and HepG2 cell lines to identify the most promising compound. Compound **18** was chosen as such with a slightly lower IC_50_ on HepG2 cells, equaling to 38.76 µM. Compound **18** also showcased the most prominent effects in the VEGFR-2 inhibition assay, with IC_50_ = 0.26 µM. However, this result was nearly three times worse than that of the reference compound—sorafenib (IC_50_ = 0.10 µM). The ADMET properties of the new structures were assessed via in vitro prediction. Compound **18** did not violate Lipinski rules, a promising feature in comparison to the reference structures, Sorafenib and Doxorubicin, which violated one and three Lipinski rules, respectively. The novel structure did not showcase any effect on cytochromes (CYP3A4, CYP1A2 and CYP3A4) important for metabolism and drug interactions in comparison to Sorafenib, which affected all three of them. Compound **18** exhibited a clearance rate slower than reference compounds, which might contribute to longer dosing intervals. Regarding toxicity, structure **18** might cause hepatotoxicity. Alternatively, this compound showcased a maximum tolerated dose higher than doxorubicin, but lower than sorafenib, while maintaining similar oral acute toxicity. Overall, the predicted ADMET profile should be considered favorable. However, other important methods of research such as apoptosis assessment, cell cycle analysis and in vitro/in vivo confirmation of ADMET properties were not explored by the authors. Combining with high IC_50_ values, it is evident that this structure requires further improvements. Lastly, Zengin et al. presented their novel 4-thiazolidinones (**3a–r**) and thiazolidinediones (**6a–o**) with a dual-targeting mechanism of activity affecting VEGFR-2 and carbonic anhydrase [64]. MCF-7 cells were utilized to analyze the novel compounds’ cytotoxic activity against cancer. Compounds **3b** and **6i** were the leading structures with their IC_50_ equaling to 21.32 and 22.33 µM, respectively. In terms of VEGFR-2-inhibiting capabilities, both novel compounds displayed promising results. However, **6i** showcased the most potent effect with its IC_50_ = 0.048 µM, which was nearly two times more promising than **3b** with IC_50_ equaling to 0.093 µM. Alternatively, compound **3b** displayed a slightly stronger effect on CA IX with its IC_50_ = 0.035 µM, outperforming both **6i** (0.041 µM) and the reference drug—acetazolamide (0.042 µM). The compounds’ safety was confirmed by analyzing their cytotoxicity against 3T3 healthy cells, with both structures showcasing visibly lesser cytotoxic effects. The in vitro prediction of ADMET properties performed by the authors showcased that new structures did not violate Veber or Lipinski rules of drug-likeness. However, more detailed predictions and in vitro/in vivo validation of the safety and bioavailability of the compounds were not performed. Taking into consideration the lack of mechanistic studies and comparatively high IC_50_ against cancer cells, the **6i** structures strongly require more in-depth research in the future.

#### 4.1.2. EGFR Inhibition

**Figure 8:**
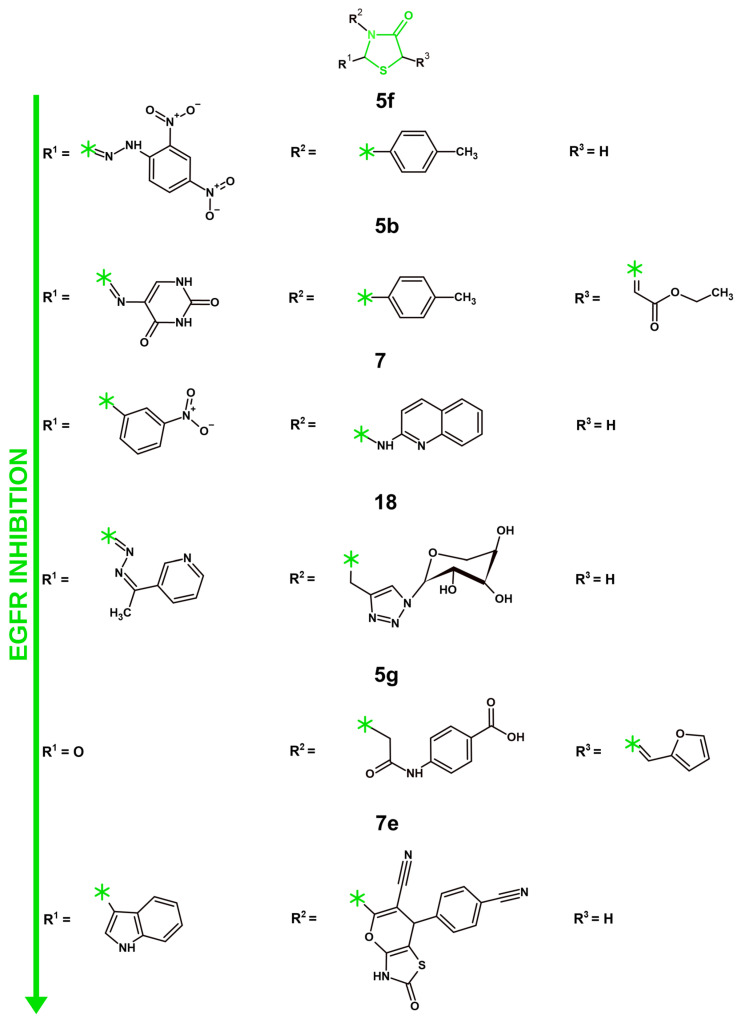
The structures of 4-TZD/TZD derivatives targeting EGFR inhibition published in 2021–2025. The compound names are preserved from their source publications for consistency.

Comparatively to thiazoles, EGFR inhibition remained a popular mechanism of activity for 4-thiazolidinones and thiazolidinediones in recent years (Table 8; Figure 8). Six articles reporting TZDs and 4-TZDs targeting EGFR in recent years have been analyzed and certain structure–activity relationships have been noted. For instance, at the C2 position of the 4-thiazolidinone core, an electron withdrawing group was present in the more active structures with **5f**, **7** featuring a nitro group and **5b** showcasing an uracil fragment. Alternatively, electron-rich indole (**7e**) or a simple oxygen (**5g**) caused the reduction in EGFR inhibitory activity. This might suggest the need for an aryl fragment with EWG (electron withdrawing group) moiety for optimal activity. The N3 position also remained highly important for EGFR inhibition. Compounds featuring simple *p*-tolyl showcased the most promising capability to inhibit EGFR. While the quinoline fragment in compound **7** was well-tolerated and contributed to activity, further increases in molecular bulk were detrimental. This was noted for compound **18**, with triazole-linked sugar moiety, and compound **7e**, with a complex multi-cyclic system. Comparatively to the C2 and N3 positions, the C5 position did not seem to significantly affect the EGFR-inhibitory activity of the analyzed structures. However, all compounds exhibited small fragments at this position; as such, the effect of bulkier structures may be more negative, and with such reasoning, it was largely avoided by authors.

In the article presented by Nafie et al., the authors analyzed a series of previously described 4-thiazolidinone derivatives linked to a quinoline moiety at the N3 atom of the 4-thiazolidinone pharmacophore [71]. As reported by the authors, the quinoline structural fragment is often utilized in anticancer research; moreover, there were several studies featuring the anticancer properties of quinoline-linked 4-thiazolidinone derivatives in the past. The most active structure was compound **7** with a nitrophenyl group, which displayed an IC_50_ equal to 7.43 µM against HCT-116 colon cancer cells. This structure was also the most potent inhibitor of EGFR, with an IC_50_ equal to 0.096 µM, as was measured by the EGFR kinase assay. Compound **7** exhibited significant induction of apoptosis with 49.76% total apoptotic cell population in comparison to the control with only 0.29% apoptotic cells. Further confirmation of the proapoptotic activities of compound **7** was provided by RT-PCR analysis, which displayed a significant increase in multiple proapoptotic proteins (p53; PUMA; Bax; and Caspases 3, 8, and 9) and a decrease in anti-apoptotic Bcl-2 protein gene expressions. Of significant importance were the results obtained from in vivo analysis. Compound **7,** at LD_50_ equal to 6 mg/kg, exhibited a tumor inhibition in a solid-Ehrlich carcinoma model in mice by 52.92%, a comparable result to 5-fluorouracil with 57.16%. The authors showcased a promising compound, **7**, which was confirmed to be a strong anticancer drug both in vitro and in vivo. However, a disadvantage should be noted, as no assessment of ADMET properties was performed for this structure. This should be a topic for further analysis in future work. Another article by Alshammari et al. displayed the results of anticancer research of novel 4-thiazolidinone derivatives with aminouracil at C2 and different substitutes at the N3 and C5 positions of 4-thiazolidinone pharmacophore [72]. The cytotoxicity assessment revealed that novel compounds, mainly **5b, 5c, 5h, 5i** and **5j**, were the most active with an IC_50_ less than 2 µM against all tested cell lines. Subsequently, those structures were chosen for further analysis of their EGFR and BRAF^V600E^ inhibition properties and respective kinase assays. While all novel structures displayed strong effects in both assays, compound **5j** was the most potent against EGFR with an IC_50_ equal to 0.087 µM, followed by **5b** (IC_50_ = 0.091 µM). It is important to note that **5b** was the most potent against BRAF^V600E^, with an IC_50_ equal to 0.093 µM, thus making it a better candidate for future research with a dual-targeting mechanism of activity. A more detailed analysis of the compounds’ effects against cancer cells was not researched by the authors despite promising initial results. Furthermore, an ADMET properties assessment and in vitro analysis against normal cells were not performed either. As such, this compound requires more in-depth research before proceeding to the next stages. In an article by Tawfeek et al., the authors synthesized novel structures with dinitrobenzene connected via a hydrazine bridge to the C2 position of the 4-thiazoldininone core [73]. After performing a cell viability assessment, the most active compound, **5f**, was identified, with 0.60 µM against MCF-7 cells. This compound also exhibited a dual-targeting mechanism of activity with the lowest IC_50_ equaling to 0.014 and 0.087 µM in CDK-2 and EGFR kinetic assays, respectively. The compounds’ proapoptotic capabilities were also confirmed, with increases in caspases 3, 8, 9 and cytochrome C concentrations comparable to or higher than the reference compound (doxorubicin). The authors did not provide results for the compounds’ effects on cell viability against normal cells, nor the assessment of the ADMET properties of their new structures. As such, the safety, pharmacokinetic and pharmacodynamic profiles of the presented compounds remains to be tested in the future. Kassem et al. presented a series of novel pyridine-4-thiazolidinone (**3–11**) and pyridine-4-thiazolidinone-triazole hybrids (**13–18**) connected to sugar moieties [74]. Among the presented compounds, the most active structure was compound **18**, a pyridine-4-thiazolidinone-triazole hybrid with glucosyl sugar, which exhibited an IC_50_ equal to 0.51 µM against MCF-7 cells. The authors also explored a dual-targeting mechanism of activity for this compound, which showcased promising IC_50_ = 0.120 and 0.180 µM for EGFR and CDK-2, respectively. The new structure exhibited a good safety profile, with IC_50_ = 84.30 µM against normal WI-38 cells. The proapoptotic effects of compound **18** in MCF-7 cells were analyzed as well. Compound **18** exhibited a notable increase in apoptotic cell population up to 40.22%. Increased concentrations of Caspase 3 and Bax were observed in MCF-7 cells treated with compound **18,** and decreased levels of anti-apoptotic Bcl-2 protein were noted as well. While the provided data exhibited promising results, more in-depth research of compound **18** effects in vivo and its ADME properties should be performed in the future. Lastly, Tiwari et al. described their novel 4-thiazolidinone derivatives targeting a mutant form of EGFR, known for its resistance to the usually applied chemotherapeutic drugs—EGFR^L858R/T790M^ [75]. Fourteen new indole–thiazolidinone derivatives were synthesized by the authors and tested against non-small cell lung cancer cell lines HCC827, NCI-H1975, and A-549 and normal immortalized lung cells—BEAS-2B. It is important to note that the HCC827 cell line contains EGFR mutation, making it highly sensitive towards EGFR inhibitors, while NCI-H1975 has already mentioned EGFR^L858R/T790M^ mutation. On the other hand, A-549 encompasses EGFR^wt^. Those cell lines were specifically chosen to study the compounds’ effects on EGFR and its resistant mutant. Among the novel structures, the compound with a cyanophenyl substitute, **7e**, displayed the lowest IC_50_ against tested cell lines, 0.23 ± 0.26 and 0.38 ± 0.19 for HCC827 and NCI-H1975, respectively. Interestingly enough, its IC_50_ against A-549 and BEAS-2B were significantly higher, 9.87 and 20.7 µM, respectively. Next, the authors analyzed the effects of **7e** on EGFR^L858R/T790M^ mutation via enzymatic activity assay and obtained promising results, with an IC_50_ equal to 0.140 µM. Unique to this article, the authors showcased **7e**’s capability to reversibly bind to the drug-resistant T790 mutant of EGFR utilizing Western blot analysis. The chosen structure displayed its capability to inhibit the phosphorylation of EGFR in a no-wash analysis and did not exhibit such effects in the wash-out experiment, confirming the reversible binding mechanism for **7e**. This is quite an important finding, because it allows the compound to overcome the C797S mutation, which causes resistance towards third-generation EGFR inhibitors, which are mainly reliant on irreversible binding to the cysteine residue. Quite possibly, it will also reduce the toxicity due to the lack of a Michael acceptor fragment, as mentioned by the authors. Compound **7e**’s effects on apoptosis were studied as well. The novel structure exhibited strong proapoptotic capabilities by showcasing 41.1% of apoptotic cells in H1975 cells in comparison to the control with 4.4% of apoptotic cells. Although the authors provided promising preliminary results of anticancer activity and selectivity towards the planned molecular target and cancer cell line, the ADMET properties of this structure were not taken into consideration in this work. As such, further research of the compound 7e effects in vivo and its ADMET profile should be performed in the future.

### 4.2. Reactive Oxygen Species (ROS) Generation

**Figure 9:**
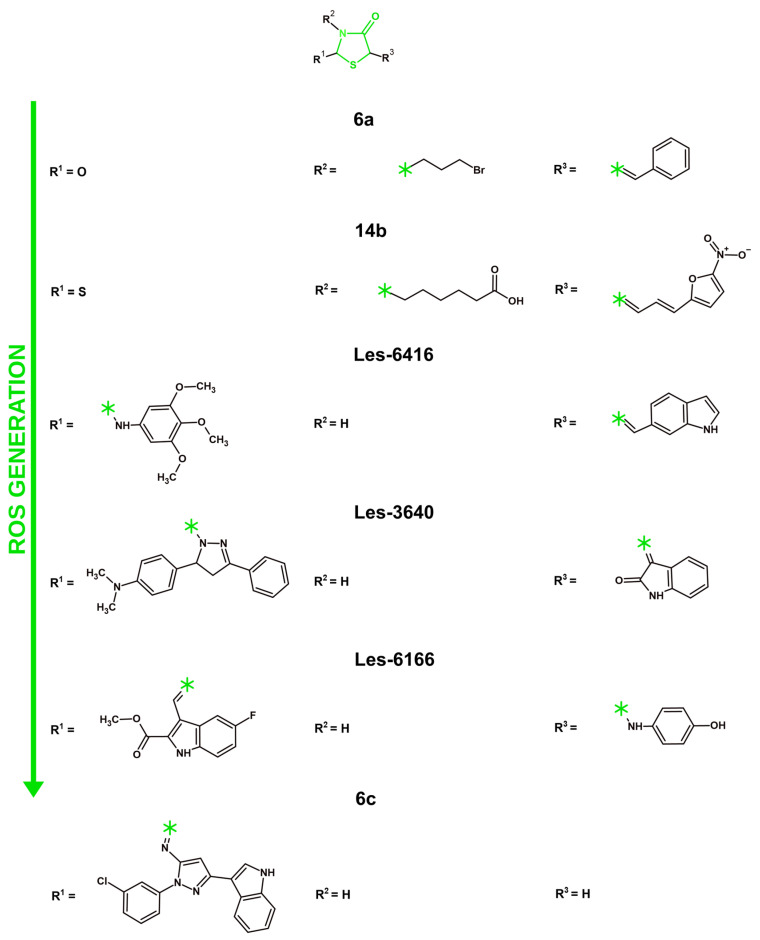
The structures of 4-TZD/TZD derivatives targeting ROS generation published in 2021–2025. The compound names are preserved from their source publications for consistency.

Reactive oxygen species generation (ROS) as an anticancer mechanism of activity was actively researched for 4-thiazolidinone and thiazolidinedione derivatives in 2021–2025 (Table 9; Figure 9). In normal conditions, healthy cells produce endogenous ROS from various oxidative processes inside the cell. To confront it, cells have antioxidant defensive mechanisms such as glutathione (GSH), superoxide dismutase (SOD), catalase (CAT), thioredoxin or peroxiredoxin. This balance allows cells to avoid damage that can be caused by ROS. However, in the case of cancer, cells are dividing and growing unconditionally, causing a spike in endogenous ROS generation. To combat it, more accumulated antioxidant resources are actively utilized. For this reason, cancer cells will have a lesser threshold of reactive oxygen species needed to cause cell death. Thus, cancer cells will be more affected by external factors causing an increase in ROS, such as anticancer drugs [115]. There are a few different mechanisms of cell death that can be caused by excessive reactive oxygen species production, such as autophagy, apoptosis or ferroptosis [115,116,117]. While ROS can induce apoptosis via both its pathways, the intrinsic one will be more actively utilized. ROS will cause the oxidation of polyunsaturated fatty acids in the mitochondrial membrane, leading to its swelling and release of cytochrome C, which inevitably leads to apoptosis. Furthermore, reactive oxygen species can also affect polyunsaturated fatty acids in the cell membrane, damaging it and activating FAS and TRAIL signaling routes of the extrinsic pathway of apoptosis. Alternatively, ROS can drive iron-dependent lipid peroxidation of the cell membrane, causing ferroptosis [118].

ROS generation was a popular mechanism of anticancer activity for TZD- and 4-TZD-containing structures, as six articles were reported in recent years. Certain structural preferences were noted for ROS generating capabilities. For instance, compounds that increased the reactive oxygen species generation to a higher degree (**6a** and **14b**) showcased chalcogen atoms (S and O) at the C2 position of the 4-TZD core. More complex and bulkier fragments at this position lead to significant loss of ROS generation. Alternatively, substituents at the N3 position also provided a significant impact on the ROS generation of analyzed compounds. Structures with N-alkyl fragments, such as **6a** (bromobutyl) and **14b** (hexanoic acid), showcased the most potent effects. On the other hand, compounds with simple hydrogen (**Les-6416**, **Les-6340**, and **Les-6166**) showcased significantly lesser potency of ROS generation. Lastly, the C5 position of the 4-TZD core also provided important insight into structural preferences for ROS generation. The simple compact and planar styryl substituent present in **6a** showcased the most potent activity. Combined with its other substituents, **6a** is a small, lipophilic molecule capable of docking into the hydrophobic pockets of enzymes, including those involved in the oxidative stress response. The exocyclic double bond of the styryl fragment allows **6a** to act as a Michael acceptor, potentially depleting cellular antioxidants such as GSH. Alternatively, **14b** has a potent “warhead” in the form of a 5-nitrofuryl moiety connected via a butadiene linker. The nitro group can directly contribute to ROS through redox cycling, likely serving as the primary driver of the compound’s capability to induce ROS generation. Although **6c** also was analyzed for its ROS generating effects, the authors did not quantify the exact values; as such, the measured effect of **6c** in terms of ROS generating capabilities remained unknown.

In an article by Szychowski et al., three 4-thiazolidinone-based derivatives were tested against SCC-15, A549, and CACO-2 cancer cells and normal BJ fibroblasts [65]. Among the novel structures, the only compound with a 4-thiazolidinone ring, **Les-3640**, showcased promise as a potential anticancer drug. This compound displayed an increase in ROS production in SCC-15, CACO-2, and BJ cells. However, it is important to note that the ROS generation caused by **Les-3640** was notably higher in the SCC-15 cell line, whereas the compound displayed an increase by 14.75%, 27.16%, and 48.77% at 0.1, 1 and 10 µM, respectively. In fibroblasts, **Les**-**3640** showcased only 25.27% increase at its highest tested concentration. However, the compound also demonstrated an evident increase in catalase and GPx expressions in SCC-15, providing certain evidence regarding the compensatory mechanisms of cancer cells, as both enzymes are an important part of the enzymatic antioxidant defense system. Alternatively, **Les-3640** exhibited a significant increase in PPARγ expression by 242.79%, while not affecting this protein’s expression in normal BJ cells. Despite that, **Les-3640** caused an increase in caspase 3 expression in all cell lines, signifying its non-selective proapoptotic effects. The ADMET properties of the analyzed structures were predicted utilizing in vitro approach. **Les-3640** did not violate Lipinski rules, nor did it showcase the capability to penetrate the blood–brain barrier, providing a certain degree of safety for the CNS. However, it showcased poor aqueous solubility, exhibiting significant lipophilic properties. Overall, the article provided an interesting perspective regarding ROS production and its compensatory mechanism; however, the selectivity of the presented compound was not adequate, and its ROS generating ability remained subpar. In another article published by Skóra et al., the authors explored the ROS-mediated anticancer mechanism of activity for four 4-thiazolidinone derivatives (Les-3166, Les-5935, Les-6009, and Les-6166) against A-549, CACO-2, and SH-SY5Y cancer cell lines and normal BJ fibroblasts [66]. Among them, the most prone to provide an increase in reactive oxygen species production was **Les-6166**. This structure amplified ROS generation after six hours of treatment up to 29% in A-549, with no significant effect on SH-SY5Y, CACO-2 and BJ cells. Alternatively, after 24 and 48 h of treatment this compound displayed a 10 to 27% increase in ROS generation in SH-SY5Y, while not affecting A-549 cells. Distressingly, the ROS production in BJ cells after 48 h of treatment with **Les-6166** was increased up to 35%. Regarding other methods of anticancer research, none of the compounds seemed to affect cell cycle in any of the tested cell lines. However, all compounds displayed an increase in caspase 3, especially in their highest tested concentrations in all cell lines, suggesting proapoptotic effects of the new compounds. Tested compounds displayed surprising results on *P. aeruginosa* biofilms. The strongest antibacterial effect was exhibited by **Les-3166**, where up to 58% of biofilm was killed at 100 µM. Alternatively, **Les-6166** did not exhibit any effect on bacteria. A significant concern regarding **Les-6166** is the lack of selectivity towards cancerous cells, even if it did exhibit a certain amount of selectivity towards human cells by not affecting bacteria. Moreover, the authors did not analyze the ADMET properties of their structure and the ROS generating effect of this structure remained at the lower level in comparison to some other compounds with this mechanism of anticancer activity. Another article featuring novel 4-thiazolidendione derivatives with ROS-mediated anticancer mechanism of activity was published by Abd Al Moaty et al. [67]. A rather unique method of synthesis was applied to create new 5-arylidene-2,4-thiazolidendione structures (**3a–e**, **4a–e**, **5a**,**c–e**, **6a**,**c–e**, and **7a**,**c**,**d**). The authors utilized ultrasound irradiation for alkylation reactions, which was more effective than traditionally applied methods in terms of selectivity, yields, and reaction rates. For example, the alkylation reactions using ultrasound were completed rapidly, within 20 to 60 min at room temperature. In contrast, traditional thermal methods, such as the reflux method used for the initial synthesis of the intermediate compounds (**3a–e**), required 3 to 15 h. The novel compounds were tested for their cytotoxic effects against Caco-2 cancer cells. Compounds **3c**, **6a** and **6e** were identified as the most active with IC_50_ equal to 4.72, 4.74 and 4.41 µM, respectively. In terms of safety, compound **6a** showcased the highest concentration, at which 100% of normal cell viability was attained, equaling to 18.94 µM. The other two structures, **3c** and **6e**, exhibited 6.07 and 10.68 µM, respectively. In terms of ROS generation, **6a** displayed an overwhelming result with a ~21-fold increase in comparison to the control, which was significantly higher than the other two compounds or the reference drug—doxorubicin, with only ~4-fold increase. Furthermore, **6a** also showcased a notable inhibiting effect on antioxidant enzymes such as SOD, aldehyde dehydrogenase 1 and GPx with 70.14, 95.84 and 87.03% of inhibition, respectively. This crucial information may suggest that **6a** increased ROS generation by significantly impairing the antioxidant system. It is also quite possible that such results may be due to a combination of both a direct increase in reactive oxygen species production and the inhibition of several crucial antioxidant enzymes. The ADMET properties of the new structures were assessed utilizing the in vitro approach. **6a** did not violate any of the Lipinski, Veber, Muegge and Ghose rules. The compound also exhibited a high level of GIT absorption, no hepatotoxicity and no effect on CYP3A4 or CYP2D6. However, **6a** exhibited the capability to penetrate the BBB, which could contribute to undesirable effects on the CNS. **6a** displayed promising results in terms of activity, safety and ADMET properties and should be further researched as a potential anticancer drug through in vivo methods. Another article featuring novel 4-thiazolidinone derivatives with ROS-induced anticancer effects was released by Dudchak et al. [68]. In their work, the authors tested five new structures with trimethoxyphenyl connected to the C2 atom of 4-thiazolidinone pharmacophore via an amine linker and various substitutes at the C5 atom of 4-thiazolidinone against the wide range of cancer cell lines presented in Table 9. The obtained data showcased that **Les-6418**, **Les-6416** and **Les-6381** were the most potent ones, with IC_50_ equaling to 2.24, 1.29 and 2.43 µM, respectively, after 72 h of treatment. However, in terms of safety, **Les-6381** exhibited less promising results, as it displayed a significant IC_50_ concentration against normal breast cells with 2.03 µM after 72 h. Alternatively, **Les-6416** was less toxic with an IC_50_ equaling to 6.66 µM. The notable antiproliferative effects of **Les-6416** were further confirmed by [^3^H]-thymidine incorporation and clonogenic assays. This compound also displayed a dose-dependent increase in ROS generation in MCF-7 cells, up to a ~3-fold difference in comparison to the control after 3 h of treatment. The authors also explored their most promising structure’s proapoptotic effects. **Les-6416** showcased 53.86% apoptotic MCF-7 cells after 24 h of treatment. **Les-6416** was confirmed to induce apoptosis via both intrinsic and extrinsic pathways, as it exhibited an increase in cell populations with activated proapoptotic caspases 3/7, 8, 9, and 10 and Bax proteins, and a decrease in mitochondrial membrane potential. An ELISA confirmed the increased concentration of two other proapoptotic proteins, p53 and cytochrome C, after treatment with **Les-6416**. An in vitro analysis was applied to assess the ADMET properties of the new structures. **Les-6416** did not violate Lipinski rules and showcased the most promising bioavailability among the new structures, which might contribute to its overall better anticancer effects in comparison to the similar **Les-6418**, which showcased lesser predicted bioavailability. Taking into consideration all data presented in this article, **Les-6416** displayed promising effects as a potential anticancer drug with its confirmed proapoptotic effects and optimal ADMET and safety evaluation. However, the compound lacks further and more in-depth analysis of its effects utilizing in vivo methods. Such analysis should be performed in the future. Lastly, Podolak et al. presented 15 new structures with 4-thiazolidinone pharmacophore and a 5-nitrofuranpropenylidene moiety as potential anticancer compounds [69]. The authors tested their novel structures against the wide range of cancer and normal cell lines displayed in Table 9. Taking into consideration the cytotoxicity results, compound **14b** was identified as the most active structure, with IC_50_ equaling to 0.85 and 6.61 µM against MCF-7 and MDA-MB-231 cell lines, respectively. A [^3^H]-thymidine incorporation assay further confirmed the potent effects of **14b** on MCF-7 and MDA-MB-231 cells’ viability and proliferation with IC_50_ equaling to 2.6 and 1.6 µM, respectively. In terms of ROS generation, **14b** significantly outperformed other tested structures and the reference compound—doxorubicin with ~10-fold increase in MDA-MB-231 cells after 1 h of treatment. **14b** exhibited a potent proapoptotic effect by increasing the total apoptotic cell population up to 29.2% in the MCF-7 cell line and up to 28.8% in the MDA-MB-231 cell line. According to the authors, an overwhelming increase in ROS generation activates the intrinsic pathway of apoptosis, which was confirmed by the decrease in mitochondrial membrane potential and an increase in the concentration of proteins characteristic to these pathways of apoptosis: cytochrome C, Bax, caspase 9 and caspase 3. The authors provided ample confirmation of the anticancer activities of compound **14b** as a strong ROS generation inducer. However, the lack of ADMET assessment and testing against normal cell lines should be noted. As such, compound **14b** requires intensive research into its safety, pharmacokinetic and pharmacodynamic properties in future studies.

### 4.3. PPARγ Activation

The peroxisome proliferator-activated receptor γ (PPARγ) is a nuclear receptor, most famously known for being a molecular target for a number of antidiabetic drugs such as rosiglitazone or pioglitazone. It plays an important part in the regulation of metabolic homeostasis. This receptor is primarily expressed in adipose tissues, but, to a lesser degree, is present in liver, muscle, breast, colon and prostate tissues as well. PPARγ agonists can improve insulin sensitivity, promote adipogenesis and cause an increase in fatty acids uptake. There is also a notable amount of evidence regarding its anticancer properties [119,120,121]. PPARγ ligands can inhibit proliferation, induce cell cycle arrest, promote cell differentiation, induce the process of programmed cell death and reduce the inflammation in cancer cells. Their activity on the cell cycle is evidenced by increased expressions of p21 and p27, and decreased expression of Cyclin D1 and E promoting arrest at the G0/G1 phase. It is important to note that PPARγ ligands can induce both intrinsic and extrinsic pathways of programmed cell death. The downregulation of Bcl-2 and upregulation of Bax, Bak, and Bcl-XL are all signs of the mitochondrial pathway of apoptosis. Another significant marker of this pathway is an increase in proline oxidase expression. Targeting this enzyme causes a notable generation of reactive oxygen species (ROS), causing significant mitochondrial damage followed by an increase in cytochrome C and caspase 9 expressions. Alternatively, some PPARγ ligands, such as rosiglitazone, displayed its capability to induce apoptosis via the extrinsic pathway, by sensitizing cancerous cells to TRAIL cytokine (TNF-related apoptosis-inducing ligand) and provide FasL ligand activation by binding to its receptor Fax. Another important aspect of the anticancer effectiveness of PPARγ ligands is their capability to shut down the signaling routes responsible for the survival and proliferation of cancer cells, mainly the PI3K/AKT/mTOR and MAPK/ERK pathways. Finally, some PPARγ ligands (Troglitazone and Rosiglitazone) exhibited the ability to inhibit the NF-kB pathway, responsible for the inflammation response [120,121]. Overall, PPARγ ligands were identified as compounds with multiple anticancer properties. There are, however, a certain number of shortcomings to this approach as well. While the vast activity of PPARγ ligands could be beneficial in cancer therapy, it also has higher risks of side effects. Moreover, not every pathway was intensely researched, and some of them were not confirmed as a direct result of PPARγ activation or inhibition.

**Figure 10:**
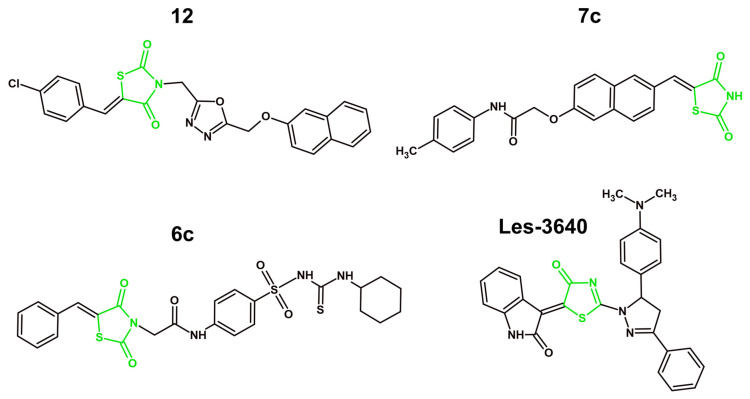
The structures of 4-TZD/TZD derivatives targeting PPARγ activation published in 2021–2025. The compound names are preserved from their source publications for consistency.

Nonetheless, in recent years, a number of articles have studied this receptor as a potential molecular target for anticancer research. Four articles in recent years focused on novel PPARγ ligands with a dual-targeting mechanism of activity (Figure 10, Table 10). 

Nazreen et al. synthesized a series of novel thiazolidinediones linked with 1,3,4-oxadiazoles affecting PPARγ and thymidylate synthase [76]. Thymidylate synthase is a crucial enzyme in such processes as DNA synthesis and repairment by transforming deoxyuridine monophosphate to deoxythymidine monophosphate. Mutations and overexpression of thymidylate synthase will cause increased and unrestrained cell division. Such well-known FDA-approved anticancer drugs as 5-fluorouracil or pemetrexed inhibit the process mediated by thymidylate synthase. Thus, it is an important target in anticancer research [122,123]. The novel compounds were analyzed for their capability to activate PPARγ receptors in comparison to rosiglitazone and pioglitazone. Among new structures, compound **12** showcased the most promising results with 78.9% transactivation activity, which was notably higher than pioglitazone (65.3%) and slightly less than rosiglitazone (83.4%). Further confirmation of the PPARγ agonistic effects of new structures was provided by an RT-PCR analysis of gene expression for this receptor. Similarly to the previous analysis, compound **12** exhibited a 2.4-fold increase in expression of PPARγ, which was considerably more than pioglitazone (1.5-fold) and marginally below the result of rosiglitazone (2.6-fold). In terms of the inhibition of thymidylate synthase, compound **12** exhibited the lowest IC_50_ required (3.2 µM). This result was even more impressive than the reference drug pemetrexed with 5.6 µM. The novel compounds were tested for their cytotoxic activity against MCF-7 and HCT-116 cancer cell lines in comparison to doxorubicin and 5-fluorouracil. Compound **12** showcased a significant inhibition of cell viability with an IC_50_ equal to 1.4 and 1.8 µM against MCF-7 and HCT-116, respectively. The ADMET properties of new structures were assessed utilizing in vitro prediction. Compound **12** exhibited promising results since it did not violate Lipinski rules, did not penetrate the BBB and exhibited high GIT absorption, likely contributed by its lipophilicity. While the authors provided adequate evidence regarding the dual-targeting mechanism of compound **12**, further in-depth research regarding the safety profile tested in vitro and/or in vivo should be performed in the future. Another article with novel dual-targeting thiazolidinediones was published by Tilekar et al. [77]. The authors synthesized novel compounds with HDAC-inhibiting and PPARγ-activating properties. The novel structures were designed as the naphthylidene thiazolidinediones derivatives, as the naphthalene fragment was present in the partial PPARγ agonist netoglitazone. This decision was derived from the evidence of lesser risks of side effects in partial agonists in comparison to the full agonists. All 25 novel structures were analyzed for their effects on PPARγ utilizing a PPARγ transactivation assay. The obtained results showcased that six structures were identified as partial PPARγ agonists (**7c**, **7i**, **7l**, **7o**, **7r**, and **7q**), with compound **7i** being the most active one (EC_50_ = 0.245 µM). Regarding their second molecular target, HDAC, new 4-thiazolidinone derivatives were analyzed against HDAC4 and HDAC8. All of them exhibited inhibiting effects on HDAC4, with twelve also inhibiting HDAC8. Compound **7w** was the most potent one in this research with IC_50_ values equal to 0.42 and 2.70 µM for HDAC4 and HDAC8, respectively, while compound **7i** inhibited only HDAC4 with an IC_50_ equal to 1.1 µM. Next, compounds that affected both targets were analyzed for their cytotoxic properties against CEM, Ramos, HL-60, HeLa, MDA-MB-231, and SH-SY5Y cancer cells, and normal HS-27 cells. Surprisingly, compound **7c** displayed the lowest IC_50_ on CEM cells with CC_50_ equal to 2.8 µM, a result comparable to the reference drug Vorinostat (CC_50_ = 2.5 µM). More importantly, the selectivity index for **7c** was equal to 14.4, the highest score among new structures. While **7c** was not the strongest inhibitor of the tested enzymes with IC_50_ values for HDAC4 and HDAC8 equaling to 1.7 and 9.0 µM, respectively, and EC_50_ for PPARγ equal to 1.22 µM, this structure was identified as the most promising compound due to its observed strong cytotoxicity and optimal safety profile, while also having sufficient effect on both molecular targets. **7c** exhibited proapoptotic effects on CEM cells; however, the cell cycle analysis did not showcase statistically significant difference in comparison to the untreated control. A Western blot analysis further confirmed the proapoptotic activity of **7c**, as this compound increased the expression of cleaved caspase 3 and suppressed the c-Myc expression. In vivo evaluation of **7c** on mice with CEM tumor xenografts showcased promising results with a reduction in tumor size by around 48% in comparison to the untreated control with no consequential loss of weight. The authors displayed an extensive work regarding their novel dual-targeting approach. Compound **7c** exhibited promising results in numerous presented analyses; however, the lack of ADMET assessment should be noted. Although the in vivo analysis provided promising results not only in terms of activity, but also in terms of safety, as **7c** did not showcase notable loss of weight in mice, the pharmacokinetic and pharmacodynamic properties of this compound remain unknown and have to be tested in the future research.

### 4.4. Tubulin Inhibition

**Figure 11:**
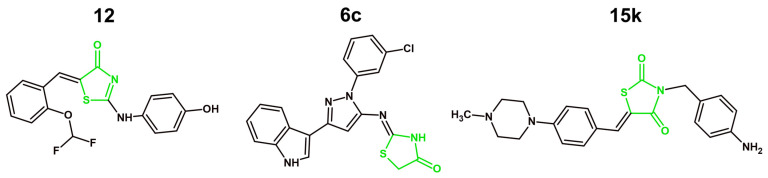
The structures of 4-TZD/TZD derivatives targeting tubulin inhibition published in 2021–2025. The compound names are preserved from their source publications for consistency.

Comparatively to thiazole derivatives, tubulin has been a popular target for novel 4-thiazolidinone derivatives in recent years as well (Table 11; Figure 11). In an article published by Rehulka et al., the authors presented 12 new structures with potential tubulin-inhibiting properties [78]. Their compounds were probed for antineoplastic activity against the range of cell lines displayed in Table 11. Of special interest are the CEM-DNR and K562-TAX cell lines which are daunorubicin and paclitaxel-resistant, respectively. The analyzed data showcased that the new 4-thiazolidinone derivatives were active against tested cell lines, with little difference between resistant and non-resistant cell lines. However, in terms of safety, compounds **10, 8, 6,** and **5** exhibited SI values of less than 2. For comparison, the selectivity indexes of potent compounds **12, 11** and **7** were over 8. A tubulin polymerization assay was applied to analyze the new structures’ effects on the tubulin protein. Similar to nocodazole, compounds **3, 10,** and **12** exhibited a tendency to inhibit tubulin polymerization with V_max_ equaling 7.7, 7.9, 5.5 and 6.8 mOD/min, respectively. For comparison, the V_max_ of the control with the vehicle substance was equal to 11.0 mOD/min. Further confirmation of the tubulin-inhibiting properties of the most promising structures was displayed by cell cycle analysis, where compounds **3, 10** and **12** arrested over 75% of the cell population in the G2/M phase, a characteristic result for this mechanism of anticancer activity. Moreover, a notable increase in the sub-G1 phase of the cell cycle was noticed as well. This suggests an increase in apoptotic cell population after treatment with new compounds. Confocal microscopy imaging also confirmed **3, 10** and **12** as potent tubulin inhibitors, since they showcased a complete disruption of the mitotic spindle. It should be noted that the ADMET properties of new structures were not assessed. While hit compound **12** showcased promising anticancer activity with confirmed tubulin-inhibiting mechanism, positive selectivity indices and cytotoxic effect against paclitaxel resistance cell line, further research into the compounds’ pharmacokinetic and pharmacodynamic properties should be assessed in the future research. Another article featuring novel tubulin inhibitors was published by Soni et al. [70]. The authors synthesized a considerable library of 34 new 4-thiazolidinones (**6a–h**) with the indole, an often-utilized fragment for tubulin inhibitors, and pyrazole, which was utilized as a linker between indole and 4-thiazolidinone. The new compounds were tested against the range of cell lines mentioned in Table 11. From the obtained data, compound **6c** was identified as the main structure with the lowest IC_50_ equal to 3.46 µM against SK-MEL-28 cells. In terms of safety, this structure showcased a promising result, when tested against Beas-2B normal cells with an SI equal to 4.59. **6c** inhibited tubulin polymerization by ~60% at 1.73 µM concentration. After treatment with **6c,** a slight increase in the G2/M phase of the cell cycle was noticed, which also serves as a marker for tubulin-inhibiting mechanism of anticancer activity. Interestingly enough, **6c** also exhibited an increase in ROS generation as was indicated by DCFDA staining and assessment. Several methods of fluorescence microscopy were applied to analyze morphological changes in the tested cells. Characteristic changes for apoptosis were spotted: cell shrinkage, nuclear fragmentation and condensation, membrane blebbing, and formation of apoptotic bodies. ADMET properties were assessed utilizing in vitro approach. Compound **6c** did not showcase any violations of Lipinski rules; it has no predicted effect on the CNS, shows promising metabolic stability and showcased incredible oral bioavailability. It also exhibited high lipophilicity and serum albumin binding. While the article displayed promising results the connections between apoptosis and either of the two mechanisms of its activity were not made. Its ROS generating capabilities were not deeply analyzed and were not quantified either. Its selectivity indices, although positive, are not significant. The compounds’ mechanism of activity and selectivity should be further analyzed in the future before continuing to further stages of drug design. Lastly, Guo et al. synthesized a series of novel thiazolidinedione derivatives with the dual-targeting mechanism of activity, aiming to inhibit the β-catenin/TCF4 interaction and polymerization of tubulin [79]. According to the authors, more than 90% of cases of colorectal cancer have a mutation in APC of β-catenin genes, that keeps their signaling pathway constantly active. β-catenin plays a crucial role in the transcriptional regulation and chromatin interactions by acting as an intracellular signal transducer. Targeting its interaction with TCF4 in the nucleus will stop the last part of the β-catenin downstream pathway, working regardless of occurring mutations. The authors modified the existing inhibitor of β-catenin/TCF4 interaction—iCRT14—by introducing various substitutes at the C5 and N3 positions of the thiazolidinedione ring. The novel compounds were analyzed for their cytotoxic properties against SW480 and HCT116 cells. Compound **15k** was identified as the most promising structure as it showcased IC_50_ values equaling to 0.86 and 1.90 µM against HCT116 and SW480 cell lines, respectively. It is a notably superior result in comparison to the reference compound iCRT14, which had 26.91 and 112.10 µM, respectively. To display the binding strength of **15k** to β-catenin, the authors applied SPR analysis. The obtained results showcased that **15k** with K_D_ of 97.5 µM exhibited around three times stronger binding than the reference compound with K_D_ of 295 µM. A co-immunoprecipitation assay further confirmed the targeted mechanism of activity, as it was revealed that **15k** significantly reduced the amount of TCF4 bound to β-catenin, which leads to decreased levels of c-Myc and cyclin D1 proteins, important for cell proliferation. Unlike the original structure, iCRT14, the compound **15k** also exhibited potent tubulin-inhibiting properties, which was ascertained by the in vitro tubulin polymerization assay and fluorescence microscopy imaging, where cells treated by the novel compound displayed disrupted microtubule network. Moreover, **15k,** at its highest tested concentration (8 µM), showcased a statistically significant difference in the G2/M phase of the cell cycle, another marker of tubulin inhibitors. The proapoptotic effects of **15k** were ascertained by flow cytometric apoptosis assessment, where **15k** exhibited statistically significant results in comparison to the untreated control. Furthermore, cleaved caspase 3 and PARP protein expressions were significantly higher than the other tested structures and control, providing even more confirmation for this compound’s potent proapoptotic effects. Overall, this article provided detailed information and confirmation of the dual-targeting mechanism of their new thiazolidinedione derivative **15k** against colon cancer cells. However, the safety profile in vitro and/or in vivo and its ADMET properties remains to be analyzed in the future as it was not assessed in the manuscript.

### 4.5. Topoisomerase Inhibition

**Figure 12:**
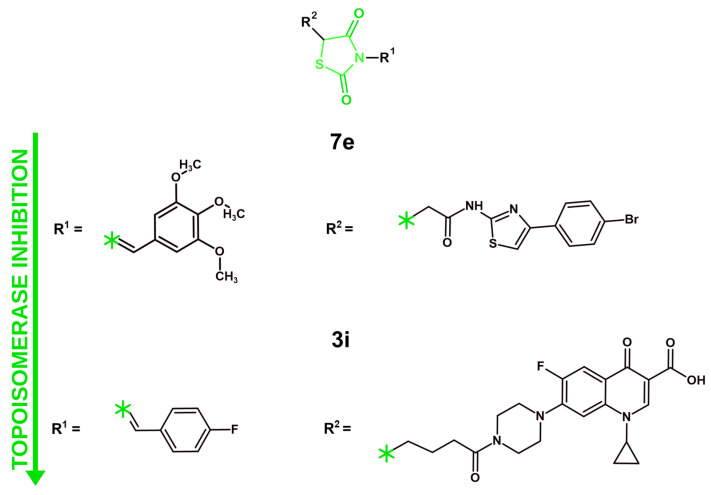
The structures of 4-TZD/TZD derivatives targeting topoisomerase inhibition (**7e** and **3i**) published in 2021–2025. The compound names are preserved from their source publications for consistency.

Inhibitors of topoisomerase enzymes have been actively utilized by anticancer compounds for a long period of time. There are two main classes of compounds targeting this mechanism of anticancer activity: topoisomerase I and topoisomerase II inhibitors. In normal conditions, topoisomerase I cuts one strand of DNA to allow rotation and relaxation of tension, while the topoisomerase II enzyme cuts both strands of DNA to pass another duplex of DNA and reseal the previously cut DNA strands. Topoisomerase I inhibitors are represented by such drugs as irinotecan or topotecan, which bind to this enzyme causing significant damage to the cell DNA during multiplication as the single strand break causes irreversible double-strand destruction. Topoisomerase II inhibitors represented by doxorubicin, daunorubicin, etoposide and mitoxantrone act similarly by creating a complex with the enzyme, forbidding the resealing of DNA after its cut, which results in irreparable DNA damage, causing the cell to trigger programmed cell death [124]. Two articles featuring novel thiazolidinediones with this mechanism of activity were reported in recent years (Figure 12) [80,81]. Both articles demonstrated new thiazolidinediones as topoisomerase I and II inhibitors. However, it is worth noting that the first article showcased better-performing compounds in terms of potency and safety, such as **7e**, in comparison to **3i**, which had notably more complex and heavier fragment at the N3 position of the TZD core. Such results may suggest that less bulky structures would be more preferable for this mechanism of activity.

In the article published by Sinicropi et al., new structures with the trimethoxybenzene moiety were linked to the C5 position and various thiazole derivatives were linked by acetamide to the N3 position of the thiazolidinedione pharmacophore [80]. The new compounds were probed for their cytotoxic activity against a few cancerous—MDA-MB-231, MCF-7, and A2058 (melanoma)—and two normal—MCF-10A and 3T3 BALB (mouse embryonic fibroblast)—cell lines. Among the novel structures, **7e** was identified as the most promising compound from this manuscript due to its significant activity against cancer cells, with MCF-7 being the most affected line with an IC_50_ equaling to 3.1 µM. Normal cells were much less affected by it, with IC_50_ values equal to 35.0 and over 100 µM for 3T3 BALB and MCF-10A cells, respectively. Further analysis into the novel compounds’ mechanism of activity showcased that **7e** inhibited both enzymes, topoisomerase I and II, while some other active structures synthesized by the authors, mainly **7c** and **7d,** exhibited inhibiting effects only for topoisomerase II. Compound **7e** also showcased proapoptotic effects via the intrinsic pathway, as was confirmed by mitochondrial destabilization, cytochrome C release, and the activation of caspases 3/7 and 9. However, the authors did not assess the novel compounds’ ADMET properties; thus, further research into the pharmacokinetic, pharmacodynamic and toxicity profiles of the compound **7e** should be performed in the future research. In the article presented by Aziz et al., the authors showcased their new structures with ciprofloxacin moiety connected to the N3 of thiazolidinedione via butyryl tail [81]. On the other side, at the C5 position various benzylidene derivatives were added. After performing a cytotoxicity assessment, compounds with smaller substitutes at the C5 position, such as **3i** with 4-fluorophenyl, displayed more promising results in comparison to the derivatives with larger radicals. Compound **3i** was the most promising among the batch with an IC_50_ equal to 25.40 µM against melanoma cells (LOX IMVI). While **3i** displayed lesser toxicity against normal cells, the difference was not significant with the IC_50_ = 45.13 µM. Similarly to the previous article, the new compound also displayed the tendency to inhibit both topoisomerases; however, it had a stronger affinity for topoisomerase I (IC_50_ = 4.77 µM) in comparison to topoisomerase II (IC_50_ = 15 µM). Further confirmation of this mechanism of activity was obtained from the cell cycle analysis, where **3i** arrested cells at the S phase, a common occurrence for topoisomerase inhibitors. **3i** also exhibited proapoptotic activity with 27.2% apoptotic cells after 24 h of treatment. Additionally, it displayed an astonishing 49-fold increase in expression of active caspase 3 and a 3-fold increase in Bax proteins in tested cancer cells. An in vitro approach was utilized to assess the ADMET properties of the new structures. Compound **3i** showcased notable lipophilicity and met most of the Lipinski rules, except for the molecular weight. Its overall predicted bioavailability was in the favorable range, as was noted by the authors. Although the authors provided an extensive analysis with confirmed mechanism of anticancer activity, the compound’s selectivity towards cancer cells remained subpar. Its cytotoxic activity also remained on the higher side with an IC_50_ over 25 µM. Thus, further research and structural optimization is required.

### 4.6. Other Targets and Mechanisms of Anticancer Activity Aimed by 4-Thiazolidinones/Thiazolidinediones Derivatives in 2021–2025

**Figure 13:**
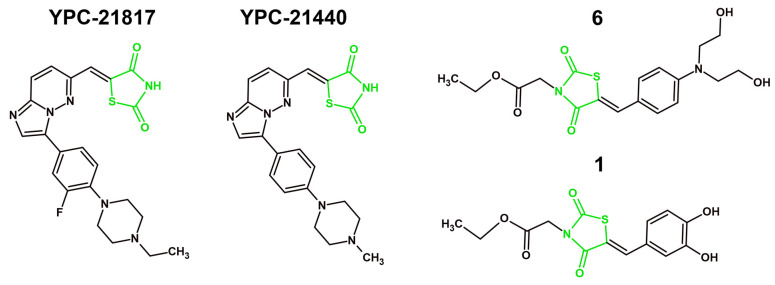
The structures of 4-TZD/TZD derivatives targeting pan-PIM kinase inhibition (**YPC-21440** and **YPC-21817**), novel BAG3 inhibitor (**6**), and its reference compound (**1**). The compound names are preserved from their source publications for consistency.

A less commonly known target for anticancer research is pan-PIM (PIM-1, 2, and 3) kinase inhibition. As of 2026, there are no FDA-approved drugs targeting this mechanism of anticancer activity, which makes this approach even more interesting for new potential drugs. PIM-1, 2, and 3 kinases are overexpressed in such types of cancer as leukemia, lymphoma and prostate cancer. PIM kinases play a significant role in mechanisms of apoptosis prevention through BAD; BIM phosphorylation; cell cycle progression through phosphorylation of p21 and p27, which promotes transition from the G1 to S phase; and protein synthesis by activating the mTOR pathway of cancer cells. Moreover, they also promote drug resistance by upregulating drug efflux pumps (ABC transporters). Given their critical role in oncogenesis, PIM kinases are significant targets for drug development. However, the overlapping substrates and redundant biological roles of PIM-1, -2, and -3 necessitate a pan-PIM inhibitory strategy. Targeting a single PIM isoform is frequently insufficient, as isoform compensation allows the tumor to circumvent the blockade. The simultaneous inhibition of all three isoforms is therefore essential to achieve significant clinical outcomes and prevent treatment resistance [125]. Sawaguchi et al. analyzed a series of hybrid 2,4-thiazolidendione derivatives with an imidazopyridazine linked to the C5 of 2,4-thiazolidinedione pharmacophore via a double bond (Figure 13) [82]. The C3 position of imidazopyridazine has various phenyl-piperazine substitutes, which provide the main difference between the tested structures. Compounds with the shortest piperazine chains exhibited the most potent activity, with **YPC-21440** (methyl chain) and **YPC-21817** (ethyl chain) exhibiting the lowest IC_50_ concentrations required for PIM-1, 2, and 3 inhibition ranging from 0.11 to 0.012 µM across all tested PIM kinases. The tested compounds exhibited potent inhibitory effects across a wide range of different types of cancer (colon, prostate, ovary, lung, breast, pancreatic, ovary, leukemia and stomach) with the IC_50_ ranging from 0.024 to 1.0 µM across all tested cell lines. **YPC-21440** and **YPC-21817** exhibited a dose-dependent reduction in phosphorylated PIM substrates Bad, 4EBP and p21, which was observed in a Western blot analysis. Furthermore, both compounds managed to arrest the cell cycle at the G1 phase, another indicator of their PIM kinase inhibitory effect. **YPC-21440** and **YPC-21817** promoted apoptosis, which was confirmed by elevated levels of caspase-3/7 and observed DNA fragmentation post-treatment. Lastly, the authors performed an in vivo analysis utilizing mouse xenograft models of leukemia (MV-4-11) and prostate cancer (PC-3). Both compounds exhibited a significant inhibition of tumor growth in the leukemia model equal to 70% for **YPC-21817** and 77% for **YPC-21440**. This, however, was not transferred to the prostate cancer model, where the experiment was stopped for **YPC-21440** due to notable weight loss up to 7.1%. Surprisingly enough, **YPC-21817** did not exhibit similar effects, while inhibiting tumor growth by 48%, making it a promising candidate for future anticancer research. However, the authors did not assess the ADMET properties of their structures. Despite the fact that **YPC-21817** did not showcase notable weight loss in the in vivo models, its pharmacokinetic and pharmacodynamic properties should be analyzed before considering further steps in the drug design.

BAG3 protein inhibition is another less actively researched target for anticancer research. It plays a significant role in the prognosis, development, and resistance of several types of cancers. Increased BAG3 levels are associated with increased proliferation, migration, survival, and invasion of cancer cells. Additionally, it promotes the expression of matrix metalloproteinase 2 and triggers the release of interleukin-8 and transforming growth factor beta [126]. This protein has a significant role in blocking apoptosis as it stabilizes anti-apoptotic proteins Bcl-2 and Mcl-1, which leads to increased resistance to apoptosis-inducing treatment [127]. The BAG3 protein is notably overexpressed in leukemia, melanoma, thyroid, breast, liver and pancreatic cancers and is often associated with poor outcomes [128]. Rugiero et al. reported a series of tested 2,4-thiazolidinediones analyzed for their activity towards BAG3 protein as the main target of their anticancer research (Figure 13) [83]. The authors utilized an in vitro approach to analyze a library of approximately 23,000 compounds, which they filtered based on chemical functions, pharmacokinetic properties and structural similarity towards the previously described reference compound **1**. The deployed filtering process aimed to obtain compounds with optimal predicted ADMET properties preferential for drug design, such as: molecular weight (<400 g/mol); the number of property or descriptor values that fall outside the 95% range of similar values for known drugs (0–5); the number of reactive functional groups, which can lead to false positive in high-throughput screening (HTS) assays and to decomposition, reactivity, or toxicity problems in vivo (0–2); the estimated number of donating hydrogen bonds (0–6); the estimated number of accepting hydrogen bonds (2–20); and the predicted octanol/water partition coefficient (2.0–6.5). Further filtering steps included checking the structures for actual combability with synthetic procedures and the commercial availability of the synthons. Finally, the structures were shape-screened for their interactions with the BAG3 protein based on reference compound **1** as the reference. Through this process, the eight most promising candidates were identified, synthesized and analyzed for their binding affinity towards the target protein via the SPR assay. Among those tested, only compound **6** exhibited better results than the reference compound, **1**. Analyzing the structural differences between the two similar compounds, the main distinction was found in the radicals connected to 5-arylidene of the 2,4-thiazolidinedione pharmacophore. The N,N-bis(2-hydroxyethyl)amino group in compound **6** seems to improve the binding affinity for the BAG3 protein (K_D_ = 6.3 nM) in comparison to compound **1** with two hydroxy groups (K_D_ = 11.1 nM). Another crucial part of the structure responsible for protein binding is ethyl ester at the N3 position of 2,4-thiazolidinone pharmacophore, since both compound **1** and **6** exhibited a significantly more potent binding affinity than compound **7** (K_D_ = 53.1 nM), which was identical to compound **6**, except for the methyl ester group at the N3 atom. Compound **6** exhibited an inhibition against A375 and HeLa cell lines with IC_50_ values equal to 19.36 and 18.67 µM, respectively. The result against HeLa cells was notably better than that displayed by compound **1**, which did not showcase a measurable IC_50_ in tested concentrations up to 50 µM. Compound **6** exhibited an increase in caspase 3 and caspase 9 concentrations signifying a pro-apoptotic activity. The Western blot analysis of the BAG3 protein displayed a slight decrease in BAG3 relative protein expression, confirming the activity of compound **6**. The authors, however, did not analyze their compound against normal cell lines, thus the safety of compound **6** remains unknown and should be analyzed in the future.

## 5. Discussion

**Figure 14:**
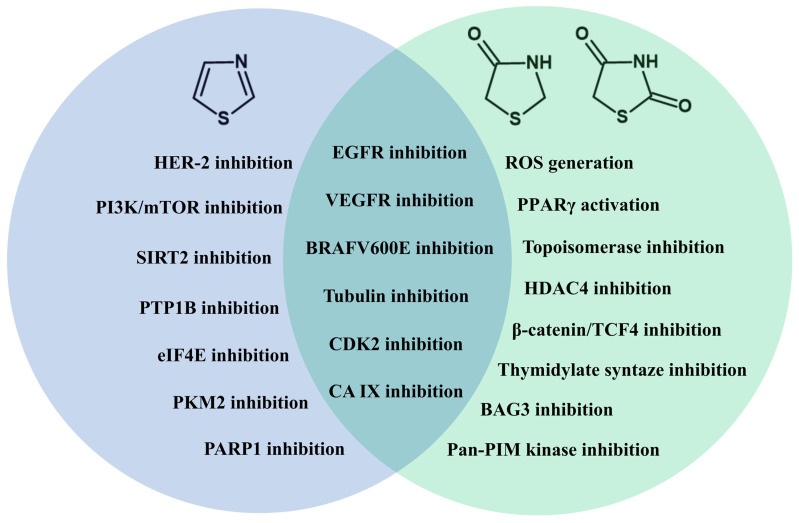
The anticancer mechanisms of activity observed for thiazole and 4-thiazolidinone/thiazolidinedione derivatives in 2021–2025.

In recent years (2021–2025) thiazoles and 4-thiazolidinones/thiazolidinediones continued to be popular structural fragments for the design of new anticancer compounds as versatile templates and indispensable parts of active compounds. The most actively studied molecular targets for both were tyrosine kinases, VEGFR-2 and EGFR, and serine/threonine protein kinases, BRAF^V600E^ and CDK-2 (Figure 14). This can be partially attributed to already existing anticancer drugs with a thiazole ring targeting tyrosine kinases, such as dasatinib, a multi-kinase inhibitor, and dabrafenib, which inhibits mutant forms of BRAF kinases, including BRAF^V600E^. Especially interesting was the work presented by Fadaly et al., where the authors created a novel thiazole derivative, **18d,** targeting the most frequent mutated forms of EGFR (EGFR^L858R^ and EGFR^L858R/T790M^), thus overcoming the chemoresistance problems often faced by previous generations of EGFR inhibitors [42]. Another promising work was published by Tiwari et al., with their indole–thiazolidinone derivative 7e, showcasing a unique approach of reverse binding to EGFR^T790M^, combating the limitations of third-generation EGFR inhibitors, related to irreversible covalent binding to the cysteine residue of EGFR^T790M^ mutation [75]. A surprisingly popular mechanism of anticancer activity for TZ and 4-TZD/TZD derivatives was tubulin inhibition (Figure 14). Taking into consideration that there are no known derivatives of those structures approved for clinical application as tubulin inhibitors suggests a potential novel approach in the field of anticancer research for those compounds. Alternatively, actively researched were PPARγ activation and ROS generation as potential mechanisms of anticancer activity for 4-TZD/TZDs (Figure 14). The popularity of PPARγ as a molecular target for novel 4-TZD/TZDs may be attributed to well-known drugs targeting PPARγ (rosiglitazone and pioglitazone), which, however, are known for a different type of activity—anti-hyperglycemic against type 2 diabetes. Despite that, the connections between anticancer activity and PPARγ have been actively explored [120,121]. Moreover, the mentioned anti-hyperglycemic drugs also displayed anticancer effects [129,130]. For those reasons authors continued to target PPARγ as a potential molecular target for antitumor research. ROS-mediated apoptosis was another mechanism of anticancer activity for 4-thiazolidinones/thiazolidinediones (Figure 14) favored by authors. The most promising compound with ROS generating capabilities was compound **6a** presented by Abd al Moaty et al. [67] with a ~21 fold increase in ROS generation in comparison to the control. Moreover, in their work the authors also established the inhibition of antioxidant enzymes caused by **6a**, essentially lowering the defensive capabilities of cancer cells to protect themselves against reactive oxygen species generation. Especially interesting were less known molecular targets, which do not have any approved drugs for clinical application as of 2025, providing an opening for new drugs to emerge. This includes PTP1B, SIRT2, PKM2, and eIF4E inhibition observed for thiazole derivatives and pan-PIM kinase and BAG3 protein inhibition for 4-thiazolidinone/thiazolidinedione derivatives (Figure 14). Among them, the most promising were PKM2 inhibitor **7d**, eIF4E inhibitor **A37** and pan-PIM kinase inhibitor **YPC-21817**, which displayed not only optimistic results of in vitro research, but also notable effects observed in vivo by reducing the tumor growth, while not causing notable weight loss during treatment.

A popular trend of creating multi-targeting anticancer compounds was observed for both TZ and 4-TZD/TZD derivatives. Approximately 30% of the analyzed works reported two or more molecular targets for their novel compounds in 2021–2025. The multi-targeting approach has a lot of potential to reduce chemotherapeutic resistance. Where a single-target drug might fail if the cell adapts, a multitargeted compound provides a “backup” mechanism to maintain efficacy. In addition to that, those anticancer mechanisms might work in synergy, with one mechanism of action potentiating another, increasing the overall potency of the compound. However, there are certain concerns with this strategy, related to increased risks of side effects due to multiple targets being affected. As such, this approach requires extensive research into the careful designing of the synthesized structures and their intensive pharmacokinetic, pharmacodynamic and safety profile assessments.

Despite the substantial progress made in recent years, several recurring flaws were observed in the analyzed manuscripts. One of the main inadequacies was the lack of ADMET properties analysis. Pharmacokinetic and pharmacodynamic profiles are of significant importance during drug design. Without the proper assessment of such properties, further advancement in drug development cannot be performed. It also should be noted that the analyzed manuscripts with an analysis of the ADMET profile of their structure present did so utilizing in vitro screening. While it is a suitable option during the early stages of drug development, a proper confirmation via in vitro and/or in vivo methods has to be performed to validate otherwise theoretical computational predictions. Another problem was the lack of safety assessment for novel compounds. As authors provide promising IC_50_ concentrations against cancer cell lines, the absence of cytotoxicity assessments against normal cell lines creates a situation where the selectivity and therapeutic potential of the analyzed structures can be questioned. This problem may also correlate with the lack of in vivo analysis for a great majority of the analyzed manuscripts, with only five of them reporting in vivo research methods. Another major concern is the relatively high IC_50_ concentrations (15~20 µM) for some of the compounds. Even during transition from in vitro research to in vivo, various issues may arise due to the more complex biological systems affecting the drug capability to reach the target. It should be noted that compounds with nanomolar IC_50_ concentrations in vitro may require micromolar doses for in vivo EC_50_ concentrations to achieve similar anticancer effects. This can be attributed to the low drug exposure to the targeted tissues. Thus, compounds with relatively high IC_50_ concentrations require even higher doses during the transition to in vivo research, which in response creates a higher chance of off-target toxicity or even accumulation of the drug in healthy tissues. This is one of the key reasons why drugs fail in Phase II/III of clinical trials despite promising preclinical data [131,132]. Another significant setback, which should be noted, is the lack of clarity in mechanistic studies. Some works have not linked their findings against the proposed molecular target and the resulting cell death, leaving the specific mode of action unclear.

While we provided the extensive research of anticancer mechanisms of activity for thiazole and 4-thiazolidinone/thiazolidinedione derivatives in 2021–2025, this work was limited to scaffold-specific analysis mainly focusing on compounds with TZ or 4-TZD/TZD rings, omitting the studies with fused heterocyclic systems and downstream derivatives where the ring system was significantly transformed during synthesis, representing a potential area for future research.

## 6. Conclusions

The systematic review of articles published in 2021–2025 confirmed that compounds with thiazole or 4-thiazolidinone/thiazolidinedione ring continued to be indispensable scaffolds for the synthesis of novel anticancer compounds. Well-established molecular targets in anticancer research, such as EGFR, VEGFR-2 and tubulin, were the main focus for researchers for all analyzed ring systems. Relaying on established over the years activity of 4-thiazolidinone/thiazolidinedione scaffolds towards PPARγ, it was one of the most popular molecular targets in recent years for compounds with the mentioned ring systems. Alternatively, seeking less known new mechanisms of anticancer activity with no approved drugs, PTP1B, SIRT2, PKM2, eIF4E inhibition for thiazoles, and pan-PIM kinase, BAG3 protein inhibition were researched for thiazoles and 4-thiazolidinone/thiazolidinediones respectively. It is worth noting the increasing popularity of the multi-targeting approach, with nearly 30% of analyzed manuscripts reporting two or more main molecular targets for their novel compounds. Ultimately, these versatile scaffolds demonstrated significant potential in anticancer drug design, as their specific mechanisms of activity can be precisely defined through targeted structural modifications.

## Figures and Tables

**Table 1:** The primary molecular targets and anticancer mechanisms of activity thiazole derivatives in 2021–2025.

№	Primary Target	Number of Articles	Ref.
1	Tubulin	5	[35,36,37,38,39]
2	EGFR (epidermal growth factor receptor)	4	[40,41,42,43]
3	VEGFR-2 (Vascular Endothelial Growth Factor Receptor 2)	3	[44,45,46]
4	CDK (cyclin-dependent kinase)-2	2	[47,48]
5	BRAF (B-Raf proto-oncogene)^V600E^	2	[40,49]
6	PI3K (phosphoinositide 3-kinase)/mTOR (Mechanistic Target of Rapamycin)	2	[43,50]
7	HER-2	2	[41,42]
8	SIRT2 (Sirtuin 2)	2	[51,52]
9	PARP (poly-ADP-ribose polymerase)-1	1	[53]
10	PTP1B (protein tyrosine phosphatase 1B)	1	[54]
11	CA (carbonic anhydraze) IX, XII	1	[55]
12	PKM2 (pyruvate kinase M2)	1	[56]
13	eIF4E (Eukaryotic Translation Initiation Factor 4E)	1	[57]
14	COX (Cyclooxygenase)-2	1	[42]

**Table 2:** The primary molecular targets and anticancer mechanisms of activity of 4-thiazolidinone/thiazolidinedione derivatives in 2021–2025.

№	Mechanism of Activity	Number of Articles	Ref.
1	VEGFR-2 inhibtion	7	[58,59,60,61,62,63,64]
2	ROS (reactive oxygen species) generation	6	[65,66,67,68,69,70]
3	EGFR inhibition	6	[61,71,72,73,74,75]
4	PPARγ (Peroxisome Proliferator-Activated Receptor gamma) activation	4	[60,65,76,77]
5	Tubulin inhibition	3	[70,78,79]
6	Topoisomerase inhibition	2	[80,81]
7	CDK-2 inhibition	2	[73,74]
8	HDAC (Histone Deacetylase)-4 inhibition	2	[58,77]
9	CA IX inhibition	1	[64]
10	BRAF^V600E^ inhibition	1	[72]
11	β-catenin/TCF4 (Transcription Factor 4) inhibition	1	[79]
12	Thymidylate synthase inhibition	1	[76]
13	Pan-PIM (Proviral Integration site for Moloney murine leukemia virus) kinase inhibition	1	[82]
14	BAG3 (Bcl-2-associated athanogene 3) inhibition	1	[83]

**Table 3:** Drug-likeness of TZ, 4-TZD and TZD heterocycles. Values were calculated via ADMETLab 3.0 software [87].

Parameter	Thiazole	4-Thiazolidinone	Thiazolidinedione	Lipinski Rule
Molecular Weight, Da	~85	~103	~117	<500
LogP	~0.54	~0.10	~0.16	<5
H-Bond Donors	0	0–1	1	<5
H-Bond Acceptors	1	2	3	<10

**Table 4:** Novel thiazole derivatives targeting EGFR inhibition.

№	The Main Compound	IC_50_ of the EGFR Inhibition, µM	Tested Cell Lines	Other Methods of Anticancer Analysis	Ref.
1	**3f**	0.089	A-549, MCF-7, Panc-1, HT-29	MTT (3-(4,5-dimethylthiazol-2-yl)-2,5-diphenyltetrazolium bromide) assay, EGFR kinase assay, BRAFV^600E^ inhibitory assay, molecular docking	[40]
2	**10a**	0.005	MCF-7, MCF-10A	MTT assay, EGFR kinase assay, cell cycle analysis, apoptosis assessment, HER-2 inhibitory assay, molecular docking, in vitro ADMET prediction	[41]
3	**18d**	0.031 (EGFR^L858R^), 0.080 (EGFR^L858R/T790M^)	MCF-7, A-549, F-180	MTT assay, EGFR kinase assays, cell cycle analysis, apoptosis assessment, molecular docking, COX-1, COX-2, HER-2 inhibitory assays, in vitro ADMET prediction	[42]
4	**6a**	0.184	MCF-7, HCT-116, HepG2, BJ-1	MTT assay, EGFR kinase assay, cell cycle analysis, apoptosis assessment, mTOR and PI3K enzymatic inhibitory assays, in vitro ADMET prediction	[43]

**Table 5:** Novel thiazole derivatives targeting VEGFR-2 inhibition.

№	The Main Compound	IC_50_ of the VEGFR-2 Inhibition, µM	Tested Cell Lines	Other Methods of Anticancer Analysis	Ref.
1	**4**	0.093	MDA-MB-231, MCF-7, MCF-10A	MTT assay, VEGFR-2 kinase assay, cell cycle analysis, apoptosis assessment, molecular docking	[44]
2	**5**	0.061	HepG2, HuH-7, THLE-2	MTT assay, VEGFR-2 kinase assay, cell cycle analysis, apoptosis assessment, measurement of gene expressions of caspase-3, Bax (Bcl-2 Associated X-protein), and Bcl-2 (B-cell lymphoma 2), AKT concentration assessment	[45]
3	**4f**	0.114	MDA-MB-231, MCF-7, PC-3, RPE-1	MTT assay, VEGFR-2 kinase assay, cell cycle analysis, apoptosis assessment, gene expression assessment of cytochrome C, Bax, Bcl-2, molecular docking, in vitro ADMET analysis	[46]

**Table 6:** The comparison of novel thiazole derivatives targeting tubulin inhibition.

№	The Main Compound	Tested Cell Lines	Cell Cycle Arrest	IC_50_ Required for Tubulin Inhibition, µM	Other Methods of Anticancer Research	Ref.
1	**5b**	MCF-7, A-549, HEK-293	G2/M	3.30	Apoptosis assessment, in vitro tubulin polymerization assay, cell cycle analysis, molecular docking, molecular dynamics	[35]
2	**7c**	HepG2, HCT-116, MCF-7, HeLa	-	2.00	MTT assay, in vitro tubulin polymerization assay, molecular docking	[36]
3	**D11**	MCF-7, A-549	G2/M	1.68	MTT assay, apoptosis assessment, cell cycle analysis, in vitro tubulin polymerization assay, confocal microscopy, molecular docking	[37]
4	**4m**	MCF-7, BT-474, A-549, MOLT-4, BxPC-3	G2/M	-	MTT assay, cell cycle analysis, in vitro tubulin polymerization assay, phase contrast microscopy, caspase 3/7 assessment, RT-PCR (Reverse-Transcription Polymerase Chain Reaction) analysis for Bax and Bcl-2, mitochondrial membrane potential evaluation via fluorescence microscopy	[38]
5	**2e**	NCI-60 cell line panel	-	7.78	Molecular docking, in vitro tubulin polymerization assay, in vitro ADMET assessment	[39]

**Table 7:** The comparison of novel 4-thiazolidinones/thiazolidinediones derivatives targeting VEGFR-2 inhibition.

№	The Main Compound	IC_50_ of VEGFR-2 Inhibition, µM	Tested Cell Lines	Other Methods of Anticancer Analysis	Ref.
1	**14c**	5.000	HT-29, A-549, MCF-7, K-562	MTT assay, VEGFR-2 inhibition ELISA, HDACs inhibition assessment, HUVEC (Human Umbilical Vein Endothelial Cell) proliferation, migration and tube formation, in vivo CAM (Chick Chorioallantoic Membrane) assay, molecular docking	[58]
2	**12a**	0.116	Caco-2, HepG2, MDA-MB-231, Vero	MTT assay, in vitro VEGFR-2 enzyme inhibition assay, scratch assay, gene expression assessment through RT-qPCR for Bcl-2, Bcl-XL, Survivin, TGF (transforming growth factor), in vitro ADMET prediction, molecular docking, molecular dynamics	[59]
3	**6c**	0.080	HCT-116, MCF-7, HepG2, Vero	MTT assay, VEGFR-2 inhibition ELISA, PPARγ binding assay, insulin secretion assessment, in vitro ADMET assessment, molecular docking	[60]
4	**5g**	0.080	HCT-116, MCF-7, HepG2, A-549,	MTT assay, VEGFR-2 kinase assay and EGFR^T790M^ kinase assays, in vitro ADMET prediction, molecular docking	[61]
5	**22**	0.079	HepG2, MCF-7, Vero	MTT assay, in vitro VEGFR-2 enzyme inhibition assay, scratch assay, apoptosis assessment, cell cycle analysis, in vitro ADMET prediction, molecular docking	[62]
6	**18**	0.260	HepG2, MCF-7, HCT-116	MTT assay, VEGFR-2 kinase assay, in vitro ADMET prediction, molecular docking	[63]
7	**6i**	0.048	MCF-7, 3T3	MTT assay, VEGFR-2 kinase assay, carbonic anhydrase IX inhibition assay, in vitro ADMET prediction, molecular docking	[64]

**Table 8:** The comparison of novel 4-thiazolidinone/thiazolidinedione derivatives targeting EGFR inhibition.

№	The Main Compound	IC_50_ of the EGFR Inhibition, µM	Tested Cell Lines	Other Methods of Anticancer Analysis	Ref.
1	**7**	0.096	HCT116, FHC	MTT assay, EGFR kinase assay, apoptosis assessment, cell cycle analysis, RT-PCR for p53, PUMA (p53 Upregulated Modulator of Apoptosis), caspases and Bcl-2, in vivo tumor analysis	[71]
2	**5b**	0.091	Panc-1, MCF-7, HT-29, A549, MCF-10A	MTT assay, EGFR and BRAFV^600E^ kinase assays, molecular docking	[72]
3	**5f**	0.087	Panc-1, MCF-7, HT-29, A549, MCF-10A	MTT assay, EGFR and CDK-2 kinase assays, assessment of caspase 3,8, 9 and cytochrome C levels, molecular docking	[73]
4	**18**	0.120	MCF-7, HepG2, WI-38	MTT assay, EGFR and CDK-2 kinase assays, cell cycle analysis, apoptosis assessment, ELISA of caspase 3, Bax, Bcl-2 concentrations, molecular docking	[74]
5	**5g**	0.140	HCT-116, MCF-7, HepG2, A-549,	MTT assay, VEGFR-2 and EGFR^T790M^ kinase assays, in vitro ADMET prediction, molecular docking	[61]
6	**7e**	0.148 (EGFR^L858R/T790M^)	HCC827, H1975, A-549, BEAS-2B	MTT assay, EGFR kinase assay, clonogenic assay, wound-healing assay, apoptosis assessment, Western blot analysis, molecular docking	[75]

**Table 9:** The comparison of novel 4-thiazolidinone/thiazolidinedione derivatives targeting ROS generation.

№	The Main Compound	ROS Generation Fold Increase in Comparison to Control	Tested Cell Lines	Other Methods of Anticancer Analysis	Ref.
1	**Les-3640**	~1.5	BJ, SCC-15, CACO-2, A549	Resazurin reduction assay, ROS generation evaluation, caspase-3 assessment, ELISA for SOD1, CAT, GPx (Glutathione Peroxidase), PPARγ and KI67 proteins expression, molecular docking, in vitro ADMET prediction	[65]
2	**Les-6166**	~1.3	BJ, A549, SH-SY5Y, CACO-2	Resazurin reduction assay, ROS generation evaluation, caspase-3 assessment, cell cycle analysis, antibacterial activity assessment, molecular docking	[66]
3	**6a**	~21	CACO-2, WI-38	MTT assay, microscopic imaging of morphological changes, ROS generation evaluation, SOD, aldehyde dehydrogenase 1 and GPx inhibitory activity assessment, molecular docking, in vitro ADMET prediction	[67]
4	**Les-6416**	-3	MCF-7, MDA-MB-231, DLD-1, HT-29, AGS, A712, T98G, MCF-10A, C8-D1A, HDFa	MTT assay, [^3^H]-thymidine incorporation assay, clonogenic assay, scratch assay, apoptosis assessment, caspases 3/7, 8, 9, 10 assessment, mitochondrial membrane potential evaluation, p53 and cytochrome C concentration ELISA, Bax concentration evaluation, fluorescence microscopy analysis of morphological changes and ROS generation, molecular docking, in vitro ADMET prediction	[68]
5	**14b**	~10	MCF-7, MDA-MB-231, HCC-1954, AGS, DLD-1, HCT-116, HT-29, SH-SY5Y, K562, Jurkat, RPMI-8866	MTT assay, [^3^H]-thymidine incorporation assay, fluorescence microscopy analysis of ROS generation, apoptosis assessment, mitochondrial membrane potential evaluation, Bax concentration assessment, assessment of caspase 9, cytochrome C and caspase 3 concentrations	[69]
6	**6c**	-	HCT116, SK-MEL-28, A549, B16-F10, BEAS-2B	MTT assay, apoptosis assessment, phase-contrast microscopy, fluorescence microscopy for ROS generation evaluation, for identifying morphological changes and apoptosis with AO (Acridine orange)/EB (Ethidium Bromide) staining, and DAPI (4′,6-Diamidino-2-Phenylindole) staining, cell cycle analysis, tubulin polymerization assay, scratch assay, molecular docking, molecular dynamics, in vitro ADMET prediction	[70]

**Table 10:** The comparison of novel 4-thiazolidinones/thiazolidinediones derivatives targeting PPARγ.

№	The Main Compound	Tested Cel Lines	Methods of Anticancer Analysis	Ref.
1	**12**	MCF-7, HCT--16	Sulforhodamine B (SRB) assay, in vitro PPARγ transactivation assay, PPARγ gene expression measurement via RT-PCR, Thymidylate synthase inhibition, cytotoxicity assessment, in vitro ADMET prediction, molecular docking	[76]
2	**7c**	CEM, Ramos, HL-60, HeLa, MDA-MB-231, SH-SY5Y, HS-27	Differential Nuclear Staining assay, in vitro PPARγ transactivation assay, HDAC-4,8 activity assay, cytotoxicity assessment, apoptosis assessment, cell cycle analysis, Western blot analysis of relative protein expressions for p53, c-Myc and cleaved caspase 3, in vivo evaluation	[77]
3	**6c, 7c**	HCT-116, MCF-7, HepG2, Vero	MTT assay, VEGFR-2 inhibition ELISA, PPARγ binding assay, insulin secretion assessment, in vitro ADMET assessment, molecular docking	[60]
4	**Les-3640**	BJ, SCC-15, CACO-2, A549	MTT assay, ROS generation evaluation, resazurin reduction assay, caspase-3 assessment, ELISA for SOD1, CAT, GPx, PPARγ and KI67 proteins expression, molecular docking, in vitro ADMET prediction	[65]

**Table 11:** The comparison of novel 4-thiazolidinones/thiazolidinediones derivatives targeting tubulin inhibition.

№	The Main Compound	Tested Cell Lines	Methods of Anticancer Analysis	Ref.
1	**12**	CCRF-CEM, CEM-DNR, K562, K562-TAX, HCT116, HCT116p53^−/−^, U2OS, BJ, MRC5	MTS (3-(4,5-dimethylthiazol-2-yl)-5-(3-carboxymethoxyphenyl)-2-(4-sulfophenyl)-2H-tetrazolium) cytotoxicity assay, BrdU (5-Bromo-2′-Deoxyuridine) incorporation analysis, tubulin polymerization assay, cell cycle analysis, confocal microscopy for β-tubulin imaging, molecular docking, molecular dynamics	[78]
2	**6c**	HCT116, SK-MEL-28, A549, B16-F10, BEAS-2B	MTT cytotoxicity assay, apoptosis assessment, phase-contrast microscopy, fluorescence microscopy for ROS generation evaluation, for identifying morphological changes and apoptosis with AO/EB staining and DAPI staining, cell cycle analysis, tubulin polymerization assay, scratch assay, molecular docking, molecular dynamics, in vitro ADMET prediction	[70]
3	**15k**	SW480, HCT116	Cell viability assay, Western blot analysis of c-MYC, cyclin D1, cyclin B1, cleaved caspase 3, PARP and β-catenin protein expressions, co-immunoprecipitation assay, SPR analysis, q-PCR (Quantitative Polymerase Chain Reaction) measurement of c-MYC and CCND1 (Cyclin D1) gene expressions, fluorescence microscopy imaging for β-catenin and tubulin, cell cycle analysis, in vitro tubulin polymerization assay, apoptosis assessment, scratch assay, molecular docking	[79]

## Data Availability

No new data were created or analyzed in this study. Data sharing is not applicable to this article.

## Abbreviations

The following abbreviations are used in this manuscript:

4-TZD	4-Thiazolidinone
ADMET	Absorption, Distribution, Metabolism, Excretion, and Toxicity
AKT	Protein Kinase B
AO	Acridine Orange
BAD	Bcl-2-associated death promoter
BAG3	Bcl-2-associated athanogene 3
Bax	Bcl-2 Associated X-protein
BBB	Blood–brain barrier
Bcl-2	B-cell lymphoma 2
BIM	Bcl-2 Interacting Mediator of cell death
BRAF	B-Raf proto-oncogene
BrdU	5-Bromo-2′-Deoxyuridine
CA	Carbonic anhydraze
CAM	Chick Chorioallantoic Membrane
CAT	Catalase
CCND1	Cyclin D1
CDK	Cyclin-dependent kinase
CNS	Central Nervous System
COX	Cyclooxygenase
CYP	Cytochrome P450
Da	Dalton
DAPI	4′,6-Diamidino-2-Phenylindole
EB	Ethidium Bromide
EGFR	Epidermal growth factor receptor
eIF4E	Eukaryotic Translation Initiation Factor 4E
ELISA	Enzyme-Linked Immunosorbent Assay
eNOS	Endothelial nitric oxide synthase
EWG	Electron withdrawing group
FDA	Federal Drug Association
G6PD	Glucose-6-Phosphate Dehydrogenase
GPx	Glutathione Peroxidase
GSH	Glutathione
HDAC	Histone Deacetylase
HER-2	Human epidermal growth factor type 2
HIF-1α	Hypoxia-Inducible Factor-1 alpha
HIV-1	Human Immunodeficiency Virus type 1
HR	Hormone receptor
HUVEC	Human Umbilical Vein Endothelial Cells
Jak	Janus kinase
MAPK	Mitogen-Activated Protein Kinase
MEK	Mitogen-activated protein kinase
MTS	3-(4,5-dimethylthiazol-2-yl)-5-(3-carboxymethoxyphenyl)-2-(4-sulfophenyl)-2H-tetrazolium
MTT	3-(4,5-dimethylthiazol-2-yl)-2,5-diphenyltetrazolium bromide
mTOR	Mechanistic Target of Rapamycin
NF-kB	Nuclear factor kappa B
PAINS	Pan-Assay Interference Compounds
PARP	Poly-ADP-ribose polymerase
PI3K	Phosphoinositide 3-kinase
PIM	Proviral Integration site for Moloney murine leukemia virus
PIP_2_	Phosphatidylinositol 4,5-bisphosphate
PIP_3_	Phosphatidylinositol (3,4,5)-trisphosphate
PKM2	Pyruvate kinase M2
PPARγ	Peroxisome Proliferator-Activated Receptor gamma
PTP1B	Protein Tyrosine Phosphatase 1B
PUMA	p53 Upregulated Modulator of Apoptosis
q-PCR	Quantitative Polymerase Chain Reaction
ROS	Reactive oxygen species
RT-PCR	Reverse-Transcription Polymerase Chain Reaction
RUNX3	Runt-domain transcription factor 3
SARS-CoV-2	Severe Acute Respiratory Syndrome Coronavirus 2
SIRT2	Sirtuin 2
SOD	Superoxide dismutase
SPR	Surface Plasmon Resonance
SRB	Sulforhodamine B
STAT	Signal Transducers and Activators of Transcription
TCF4	Transcription Factor 4
TGF	Transforming Growth Factor
TZ	Thiazole
TZD	Thiazolidinedione
VEGF-A	Vascular endothelial growth factor A
VEGFR-2	Vascular Endothelial Growth Factor Receptor 2
